# Control of glutamate release by complexes of adenosine and cannabinoid receptors

**DOI:** 10.1186/s12915-020-0739-0

**Published:** 2020-01-23

**Authors:** Attila Köfalvi, Estefanía Moreno, Arnau Cordomí, Ning-Sheng Cai, Victor Fernández-Dueñas, Samira G. Ferreira, Ramón Guixà-González, Marta Sánchez-Soto, Hideaki Yano, Verònica Casadó-Anguera, Rodrigo A. Cunha, Ana Maria Sebastião, Francisco Ciruela, Leonardo Pardo, Vicent Casadó, Sergi Ferré

**Affiliations:** 1 CNC-Center for Neuroscience and Cell Biology, University of Coimbra, 3004-504 Coimbra, Portugal; 2 Department of Biochemistry and Molecular Biomedicine, Faculty of Biology, and Institute of Biomedicine, University of Barcelona, 08028 Barcelona, Spain; 3 Laboratori de Medicina Computacional, Unitat de Bioestadística, Facultat de Medicina, Universitat Autònoma de Barcelona, 08193 Bellaterra, Spain; 4 Integrative Neurobiology Section, National Institute on Drug Abuse, Intramural Research Program, National Institutes of Health, Baltimore, MD 21224 USA; 5 Unitat de Farmacologia, Departament Patologia i Terapèutica Experimental, Facultat de Medicina, IDIBELL, Universitat de Barcelona, L’Hospitalet de Llobregat, Spain; 6 Institut de Neurociències, Universitat de Barcelona, Barcelona, Spain; 7 Faculty of Medicine, University of Coimbra, Coimbra, Portugal; 8 Instituto de Farmacologia e Neurociências, Faculdade de Medicina, Universidade de Lisboa, Lisbon, Portugal; 9 Instituto de Medicina Molecular, Faculdade de Medicina, Universidade de Lisboa, Lisbon, Portugal

**Keywords:** Adenosine A_2A_ receptor, Cannabinoid CB_1_ receptor, GPCR heteromers, Adenylyl cyclase, Glutamate transmission, Striatum

## Abstract

**Background:**

It has been hypothesized that heteromers of adenosine A_2A_ receptors (A2AR) and cannabinoid CB_1_ receptors (CB1R) localized in glutamatergic nerve terminals mediate the integration of adenosine and endocannabinoid signaling involved in the modulation of striatal excitatory neurotransmission. Previous studies have demonstrated the existence of A2AR-CB1R heteromers in artificial cell systems. A dependence of A2AR signaling for the Gi protein-mediated CB1R signaling was described as one of its main biochemical characteristics. However, recent studies have questioned the localization of functionally significant A2AR-CB1R heteromers in striatal glutamatergic terminals.

**Results:**

Using a peptide-interfering approach combined with biophysical and biochemical techniques in mammalian transfected cells and computational modeling, we could establish a tetrameric quaternary structure of the A2AR-CB1R heterotetramer. This quaternary structure was different to the also tetrameric structure of heteromers of A2AR with adenosine A_1_ receptors or dopamine D_2_ receptors, with different heteromeric or homomeric interfaces. The specific quaternary structure of the A2A-CB1R, which depended on intermolecular interactions involving the long C-terminus of the A2AR, determined a significant A2AR and Gs protein-mediated constitutive activation of adenylyl cyclase. Using heteromer-interfering peptides in experiments with striatal glutamatergic terminals, we could then demonstrate the presence of functionally significant A2AR-CB1R heteromers with the same biochemical characteristics of those studied in mammalian transfected cells. First, either an A2AR agonist or an A2AR antagonist allosterically counteracted Gi-mediated CB1R agonist-induced inhibition of depolarization-induced glutamate release. Second, co-application of both an A2AR agonist and an antagonist cancelled each other effects. Finally, a CB1R agonist inhibited glutamate release dependent on a constitutive activation of A2AR by a canonical Gs-Gi antagonistic interaction at the adenylyl cyclase level.

**Conclusions:**

We demonstrate that the well-established cannabinoid-induced inhibition of striatal glutamate release can mostly be explained by a CB1R-mediated counteraction of the A2AR-mediated constitutive activation of adenylyl cyclase in the A2AR-CB1R heteromer.

## Background

Adenosine and endocannabinoids, such as anandamide and 2-arachidonylglycerol (2-AG), are very ubiquitous non-classical neurotransmitters that modulate the transmission ensured by other more classical neurotransmitters. In the striatum, the modulatory role of adenosine and endocannabinoids converge in excitatory synapses, where their signaling is integrated by G protein-coupled receptors (GPCRs) localized in glutamatergic nerve terminals [1]. Adenosine is produced from the conversion of ATP (by ectonucleotidases) that is co-released with glutamate from the nerve terminals and from astrocytes [1, 2]. Adenosine can then bind and activate adenosine receptors of the A_1_ or A_2A_ subtype (A1R or A2AR, respectively) localized presynaptically, promoting inhibition or facilitation of glutamate release, respectively [1–3]. On the other hand, endocannabinoids are produced “on demand” from endocannabinoid precursors by the action of enzymes localized in the postsynaptic plasma membrane [4]. One of the best studied functions of endocannabinoids is “retrograde signaling” with stimulation of presynaptic cannabinoid CB_1_ receptors (CB1R) and the consequent inhibition of neurotransmitter release [4].

We have previously hypothesized that adenosine and endocannabinoids exert a fine-tune modulation of striatal glutamate release from striatal glutamatergic terminals, by which low adenosine plus high endocannabinoid tone would produce the weakest, while high adenosine plus low endocannabinoid tone would produce the strongest glutamate release [1]. We also hypothesized that this fine-tune modulation depends, not only on the adenosine and endocannabinoid tone, but on the ability of specific subtypes of adenosine and cannabinoid receptors to form heteromers, mainly A1R-A2AR and A2AR-CB1R heteromers, and on their unique biochemical properties (reviewed in [1]). A1R-A2AR heteromers have been characterized both functionally and structurally, but some inconsistencies remain about the functional properties of A2AR-CB1R heteromers and even about their existence in striatal glutamatergic terminals (see below).

The A1R-A2AR heteromer acts as a concentration-dependent switch that mediates the adenosine control of striatal glutamatergic transmission [3]. Since adenosine binds with higher affinity to A1R than to A2AR [5], low concentrations of adenosine inhibit glutamate release by activating the Gi-coupled A1R [3]. On the other hand, high concentrations of adenosine produce the opposite effect, by activating the Gs-coupled A2AR, which promotes glutamate release by activating adenylyl cyclase (AC)-PKA signaling and allosterically counteracting A1R signaling within the A1R-A2AR heteromer [3, 6]. The quaternary structure of the A1R-A2AR heteromer has been recently proposed and shown to be heterotetrameric, constituted by homodimers of A1R and A2AR coupled to their cognate G proteins [6]. This is similar to the also recently described quaternary structure of the A2AR-dopamine D_2_ receptor (D2R) heteromer [7], which is localized postsynaptically in the striatum [8].

It has been recently demonstrated that the A2AR-D2R heteromer forms part of functional complexes that include A2AR-D2R heterotetramers, constituted by A2AR and D2R homodimers with their respective cognate Gs and Gi proteins, and AC subtype AC5 [7]. The quaternary structure of these complexes is stabilized by specific interactions between transmembrane domains (TMs) of the receptors, which determine the homomeric and heteromeric interfaces in the A2AR-D2R heterotetramer, as well as between TMs of the receptors and TMs of AC5 [7]. In addition, interactions between intracellular domains play a significant role in the stabilization of the complex, namely a strong electrostatic interaction between the C-terminal domain of the A2AR (A2AR-CT) and the intracellular end of TM 5 of the D2R [9–12] and between the N-terminal domain (NT) of AC5 and βγ-subunits of the G proteins [13]. The predicted quaternary structure of the A2AR-D2R heterotetramer in complex with AC5 provided the frame for the canonical Gs-Gi antagonistic interaction at the AC level [7]. This canonical interaction implies the ability of an activated Gi-coupled receptor to inhibit AC activation by a Gs-coupled receptor [14] and requires the simultaneous respective interaction of the Ras-GTPase domain of the α-subunits of the Gs and Gi proteins with the C2 and C1 catalytic domains of AC [15].

Importantly, the heteromeric and homomeric interfaces of the A1R-A2AR heterotetramer were found to be different from those of the A2AR-D2R heterotetramer [6, 7]. The consequent different conformation of the A1R-A2AR heterotetramer was associated with its inability to sustain a canonical Gs-Gi antagonistic interaction at the AC level [6]. Particularly striking was the involvement of A2AR-CT, because its deletion enabled the canonical Gs-Gi antagonistic interaction by the A1R-A2AR heteromer. On the other hand, contrary to the A2AR-D2R heterotetramer, A2AR-CT deletion did not disrupt A1R-A2AR heteromerization, indicating its lack of involvement on the stabilization of the quaternary structure of the A1R-A2AR heterotetramer [6]. These results also indicated that the ability of A1R in the A1R-A2AR heteromer to mediate inhibition of glutamate release in the striatal glutamatergic terminals was not dependent on the canonical Gs-Gi antagonistic interaction at the AC level and, therefore, on the inhibition of A2AR-mediated AC-PKA signaling. Instead, it would be most probably dependent on the classical inhibitory effect of βγ-subunits on presynaptic calcium channels [16, 17].

The ability of A2AR and CB1R to heteromerize was first suggested from results obtained in artificial cell systems using biophysical techniques [18]. In the same study, signaling experiments performed in a neuroblastoma cell line indicated the existence of the conventional G protein coupling for both receptors and a dependence on A2AR signaling for the expression of the Gi-mediated inhibition of AC activity by CB1R agonists [18]. As expected from the canonical interaction at the AC level, a CB1R agonist could counteract an A2AR agonist-induced AC activation. But, A2AR blockade also counteracted the ability of a CB1R agonist to inhibit forskolin-induced AC activation [18]. It was then suggested that activation of A2AR in the A2AR-CB1R heteromer allows the effective coupling of CB1R to Gi proteins and, consequently, that CB1R signaling is entirely dependent on A2AR signaling [18]. Strong evidence for physical interactions between A2AR and CB1R in striatal glutamatergic terminals was afterwards reported, including a robust co-localization and co-immunoprecipitation [19]. In the same study, an A2AR agonist significantly decreased the potency of a CB1R agonist to inhibit striatal glutamate release and presynaptic corticostriatal glutamatergic transmission [19]. More in line with the expected dependence on A2AR activation within the A2AR-CB1R heteromer, some studies also found evidence for counteraction of CB1R-mediated corticostriatal transmission by A2AR antagonists [20, 21].

Although apparently incompatible with the involvement of a single population of A2AR, forming heteromers with CB1R, similar effects of A2AR agonists and antagonists have also been obtained with the A2AR-D2R heteromer and found to depend on allosteric interactions that depend on its tetrameric structure. When either an A2AR agonist or an A2AR antagonist binds to the orthosteric sites of the A2AR homodimer in the A2AR-D2R heterotetramer, they both produce an allosteric decrease in the affinity and efficacy of D2R ligands [12]. On the other hand, when an agonist and an antagonist bind simultaneously to the two orthosteric sites of the A2AR homodimer, they counteract each other’s effects [12]. At the biochemical level, a negative allosteric interaction between orthosteric A2AR agonists and antagonists in an A2AR homodimer could be demonstrated in dissociation kinetic binding experiments of a radiolabeled A2AR antagonist *versus* A2AR agonists and antagonists, including the non-selective antagonist caffeine [12]. At the behavioral level, these homomeric allosteric interactions could better explain the psychomotor stimulant effects of caffeine and selective A2AR antagonists than the classically assumed competitive antagonism between A2AR antagonists and endogenous adenosine for the same orthosteric site [22, 23]. Thus, the allosteric homomeric interactions between A2AR agonists and antagonists predicted a counterintuitive counteraction of a high locomotor depressant dose of an A2AR antagonist in the rat with an also locomotor depressant dose of an A2AR agonist in the rat [22].

There is therefore a significant amount of evidence that supports the existence of functional A2AR-CB1R heteromers in the striatal glutamatergic terminals with similar structural and biochemical characteristics to those of the A2AR-D2R heteromers and that they constitute a main population of CB1R that play an essential role in striatal synaptic transmission and plasticity [24–28]. However, in a recent study, A2AR-CB1R complexes were identified by the proximity ligation assay in the mouse striatum and suggested to represent A2AR-CB1R heteromers localized postsynaptically in GABAergic striatopallidal neurons, but not in corticostriatal terminals. This conclusion was based on results obtained in mice with differential genetic blockade of CB1R in forebrain GABAergic neurons *versus* dorsal telencephalic glutamatergic neurons [29]. In addition, parallel experiments in conditionally immortalized striatal neuroblasts suggested that A2AR-CB1R heteromers signal by Gq protein coupling, but not through Gs and Gi proteins [29]. In view of these apparently controversial results, the goals of the present study were, first, to establish the quaternary structure and biochemical properties of the A2AR-CB1R heteromer and to compare them with those of the A1R-A2AR and A2AR-D2R heterotetramers. Second, we aimed at establishing the localization, biochemical properties, and functional significance of the A2AR-CB1R heteromers in the striatal glutamatergic terminals.

## Results

### A2AR and CB1R in the A2AR-CB1R heteromer couple to their respective cognate Gs and Gi proteins in HEK-293T cells

The complemented donor-acceptor resonance energy transfer (CODA-RET) assay was first used to analyze the preferred functional G protein coupling of A2AR and CB1R in the A2AR-CB1R heteromer, in transiently transfected HEK-293T cells. In this assay, two complementary halves of the bioluminescent protein *Renilla* luciferase (Rluc8 variant; nRluc and cRluc) are separately fused to two different GPCR units (protomers) putatively able to oligomerize, and a fluorescent protein, the mVenus variant of the yellow fluorescence protein (YFP), is fused to a Gα protein subunit. Ligand-induced changes in CODA-RET measurements imply, first, a successful complementation of Rluc and, therefore, oligomerization of the corresponding protomers. Second, although CODA-RET does not provide an estimate of the degree of oligomerization (affinity or stoichiometry), it represents the reading of a specific G protein activation through the GPCR heteromer [30–32]. A2AR was fused to cRluc, and CB1R was fused to nRluc and co-transfected with either Gαs, Gαi1 or Gαq fused to YFP. Concentration-response curves of ligand-induced changes in BRET were then determined in the presence of different concentrations of the non-selective adenosine receptor agonist NECA or the selective CB1R agonist CP55940. A concentration-response curve with NECA could only be obtained when the cells were transfected with Gαs-YFP (EC_50_ values, in mean ± S.E.M: 0.35 ± 0.08 μM; *n* = 5 with triplicates), but not with Gαi1-YFP or Gαq-YFP (Additional file 1: Figure S1a; Additional file 2: Data values 1). On the other hand, a concentration-response with CP55940 could only be obtained when the cells were transfected with Gαi-YFP (EC_50_ values, in mean ± S.E.M: 1.05 ± 0.39 μM; *n* = 5 with triplicates), but not with Gαs-YFP or Gαq-YFP (Additional file 1: Figure S1b; Additional file 2: Data values 1). A positive control of Gq coupling by a GPCR heteromer was obtained with the previously reported serotonin 5-HT_2A_ receptor (5-HT2AR)-D2R heteromer [33, 34]. A concentration-response curve was obtained with serotonin, but not dopamine, when the cells were transfected with 5-HT2AR fused to cRluc, D2R fused to nRluc and Gαq-YFP (EC_50_ values, in mean ± S.E.M: 0.21 ± 0.05 μM; *n* = 5 with triplicates) (Additional file 1: Figure S1c; Additional file 2: Data values 1). These results indicate that within the A2AR-CB1R heteromer, A2AR and CB1R preferentially couple to their respective cognate Gs and Gi proteins. Nevertheless, the results do not discard the possible coupling to Gq by A2AR and CB1R in the A2AR-CB1R heteromer in another cellular environment, such as in the conditionally immortalized striatal neuroblasts and in the striatum, in the GABAergic striatopallidal neurons or astrocytes [29].

**Fig. 1:**
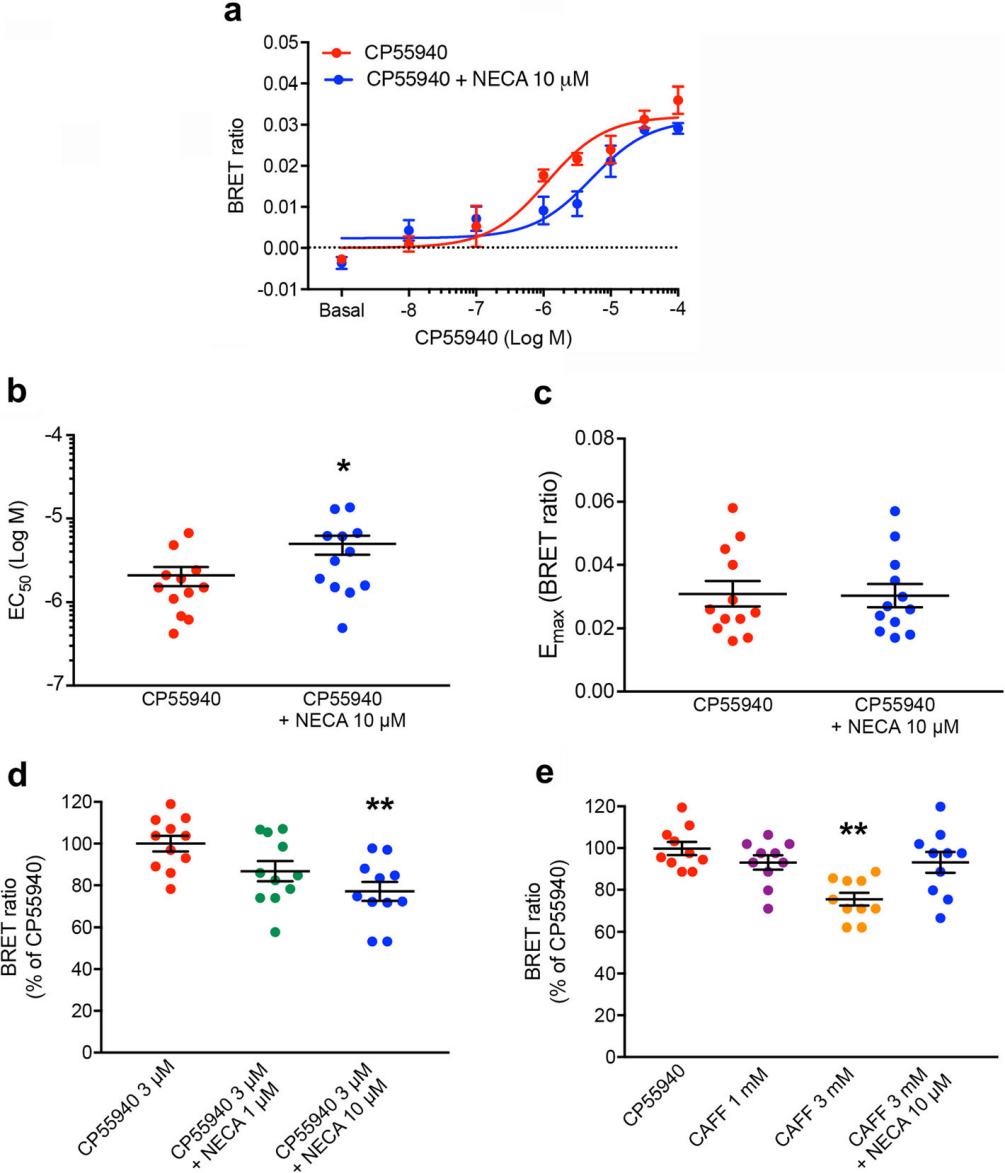
Modulation by A2AR ligands on CB1R-mediated G protein activation in the A2AR-CB1R heteromer. **a–e** CODA-RET experiments, where two complementary halves of Rluc (cRluc and nRluc) are respectively fused to the A2AR and CB1R and YFP is fused to the α-subunit of Gi. HEK-293T cells were transiently transfected with cDNAs of A2AR-cRluc (3.33 μg), CB1R-nRluc (1.67 μg), Gαi1-YFP (5 μg), and non-fused β1 and γ2 subunits (4.5 μg and 5 μg, respectively). **a** Concentration-response curves of the effect of the selective CB1R agonist CP55940 on the ligand-induced BRET changes, which are determined by changes in the interaction of the A2AR-CB1R heteromer with Gi, in the presence (blue plot) and absence (red plot) of the non-selective adenosine agonist NECA (10 μM). Data are means ± S.E.M. of triplicate BRET ratio values of a representative experiment. **b**, **c** EC_50_ and *E*_max_ values of 12 independent experiments performed in triplicate, expressed as means ± S.E.M.; the EC_50_ and *E*_max_ values were obtained by non-linear regression fitting to a sigmoidal concentration-response curve and analyzed statistically with a paired *t*-test (*: *p* < 0.05, compared with the absence of NECA). **d**, **e** Modification by NECA (1 and 10 μM), caffeine (CAFF, 1 and 3 mM) and NECA (10 μM) plus caffeine (3 mM) on the effect of an EC_50_ concentration of CP55940 (2 μM); values are means ± S.E.M. (*n* = 10–11 with triplicates) of the percentage of the effect of CPP55940 alone and analyzed statistically with repeated measures ANOVA, followed by Dunnett’s multiple comparison test (**: *p* < 0.01, compared with CPP55940 alone)

Remarkably, NECA (10 μM) produced a shift to the right in the concentration-response curve of CP55940 (Fig. 1a), with a significant increase in EC_50_ values (Fig. 1b) and no significant difference in the *E*_max_ values (Fig. 1c). These results represent a readout of a negative allosteric interaction between two orthosteric agonists within the A2AR-CB1R heteromer, by which an A2AR agonist decreases the potency of a CB1R agonist-mediated Gi protein activation. If the A2AR-CB1R heteromer would exhibit a tetrameric structure similar to the A2AR-D2R heterotetramer, we could expect the same allosteric interaction with A2AR agonists and antagonists and their counteracting effect when simultaneously applied (see “Background” and ref. [12]). In fact, both NECA and caffeine produced a concentration-dependent decrease of the change in BRET ratio values induced by an EC_50_ concentration of CP55940 (2 μM; Fig. 1d, e). In addition, effective concentrations of NECA (10 μM; Fig. 1d) and caffeine (3 mM; Fig. 1e) became ineffective when co-applied (Fig. 1e). These results therefore recapitulate allosteric interactions within the A2AR-D2R heterotetramer and, therefore, suggest an also tetrameric structure of the A2AR-CB1R heteromer.

We also analyzed the possible opposite allosteric interaction by which a CB1R agonist could modify the potency of an A2AR agonist-mediated Gs protein activation (cells transfected with Gαs-YFP). CP55940 did not produce a significant shift in the concentration-response curve of NECA. EC_50_ values of the concentration-response curves of NECA in the absence and presence of CP55940 (10 μM) were, in mean ± S.E.M, 0.52 ± 0.23 μM and 0.57 ± 0.13 μM, respectively (paired *t* test: *p* > 0.05; *n* = 5 with triplicates); *E*_max_ values in the absence and presence of CP55940 (10 μM) were, in mean ± S.E.M, 0.009 ± 0001 and 0.009 ± 0.001 BRET ratio units (BRU), respectively (paired *t* test: *p* > 0.05; *n* = 5 with triplicates) (Additional file 1: Figure S2; Additional file 2: Data values 2). These results indicate the existence of a unidirectional allosteric modulation between orthosteric ligands in the A2AR-CB1R heteromer.

### The A2AR-CB1R heteromer has a different quaternary structure compared to the A2AR-D2R and the A1R-A2AR heterotetramers

The possible heterotetrameric structure of the A2AR-CB1R heteromers was further evaluated in HEK-293T transfected cells by a BRET experiment based on the double complementation of both BRET bioluminescent and fluorescent proteins [12, 31], with complementary halves of Rluc and YFP separately fused to different protomers of A2AR and CB1R. As shown in Additional file 1: Figure S3, significantly higher BRET values could be obtained with co-transfection of A2AR-cRluc, A2AR-nRluc, CB1R-cYFP, and CB1R-nYFP, as compared with controls, where the A2AR or CB1R constructs were substituted by dopamine D_1_ receptor (D1R) constructs, D1R-cRluc and D1R-nRluc or D1R-cYFP and D1R-nYFP.

**Fig. 2:**
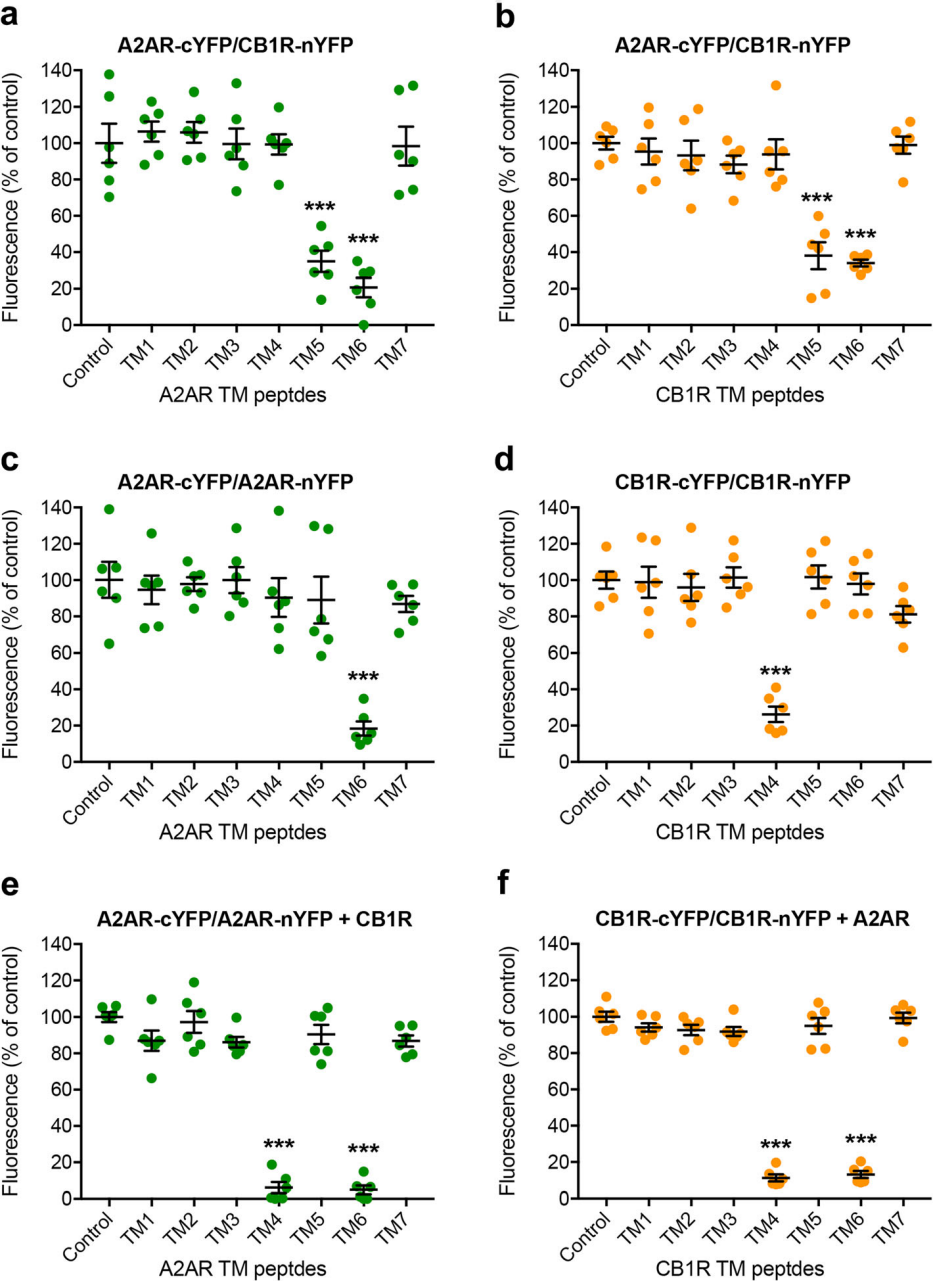
Heteromeric and homomeric interfaces in the A2AR-CB1R heterotetramer. **a–f** BiFC experiments with synthetic peptides with the amino acid sequence of all TMs of A2AR and CB1R. HEK-293T cells were transiently transfected with **a**, **b** cDNAs of A2AR and CB1R separately fused to complementary halves of YFP (A2AR-cYFP and CB1-nYFP; 6 μg in both cases); **c**, **e** cDNAs of two different molecules of A2AR separately fused to complementary halves of YFP (A2AR-cYFP and A2AR-nYFP; 3 μg in both cases) without (**c**) or with (**e**) co-transfection with cDNA of non-fused CB1R (1 μg); **d**, **f** cDNAs of two different molecules of CB1R separately fused to complementary halves of YFP (CB1R-cYFP and CB1R-nYFP; 3 μg in both cases) without (**d**) or with (**f**) co-transfection with cDNA of non-fused A2AR (1 μg). Cells were treated for 4 h with medium (control) or 4 μM of indicated TM peptides (numbered 1–7). Values are means ± S.E.M. (*n* = 6 with triplicates in all the experiments) of the percentage of the fluorescence in the control group and analyzed statistically with repeated measures ANOVA, followed by Dunnett’s multiple comparison test (***: *p* < 0.001, compared with control)

Bimolecular fluorescence complementation (BiFC) experiments with synthetic interfering peptides were then performed to elucidate the homomeric and heteromeric interfaces of the heterotetramer, which is the same strategy that was used to reveal the precise quaternary structure of the A1R-A2AR and A2AR-D2R heterotetramers [6, 7]. While BiFC complex formation under in vitro conditions (purified complementary fluorescent proteins) has been considered to be essentially irreversible [35], several studies, including our own on GPCR heteromers, indicate that under in vivo conditions (live cell preparations) BiFC complex formation can be reversible [6, 7, 31, 36–38]. Complementary halves of YFP were separately fused to A2AR (A2AR-cYFP) and CB1R (CB1R-nYFP) or two different molecules of A2AR (A2AR-nYFP and A2AR-cYFP) or CB1R (CB1R-nYFP and CB1R-cYFP), in the absence and presence of synthetic peptides with the amino acid sequence of all possible TMs of both receptors fused to the cell-penetrating HIV transactivator of transcription (TAT) sequence (TMs are abbreviated TM 1, TM 2,…; TM peptides are abbreviated TM1, TM2,…). We could first demonstrate the selective interference of BiFC of A2AR-cYFP and CB1R-nYFP with TM5 and TM6 of both A2AR and CB1R (Fig. 2a, b). These results point to the involvement of TM 5/6 in the A2AR-CB1R heteromer interface. Notably, the previously studied heteromer interface of A1R-A2AR was also TM 5/6 [6], whereas the heteromer interface of A2AR-D2R was found to be TM 4/5 [7]. BiFC of A2AR-nYFP and A2AR-cYFP (in the absence of CB1R) was only significantly reduced by TM6 (Fig. 2c), as previously reported [7], and BiFC of CB1R-nYFP and CB1R-cYFP (in the absence of A2AR) was only reduced by TM4 (Fig. 2d). These results indicate that TM 6 forms the A2AR interface and TM 4 forms the CB1R interface for homodimerization, when each receptor is expressed alone. Significantly, the interface for both CB1R-CB1R and A2AR-A2AR homodimers changed in the presence of the other non-fused molecularly different receptor (Fig. 2e, f). Thus, TM4 and TM6 of A2AR reduced BiFC of A2AR-cYFP and A2AR-nYFP in the presence of non-fused CB1R (Fig. 2e) and TM4 and TM6 of CB1R reduced BiFC of CB1R-cYFP and CB1R-nYFP in the presence of non-fused A2AR (Fig. 2f). The results in Fig. 2 are expressed as means ± S.E.M. of the percentage of the fluorescence in the control group, where control values (without interfering peptides) were always between 12,000 and 15,000 relative fluorescence units (RFU; value that depends on the gain setting in the measurement equipment). Non-transformed data show no significant change in fluorescence units upon co-transfection with a non-fused receptor.

**Fig. 3:**
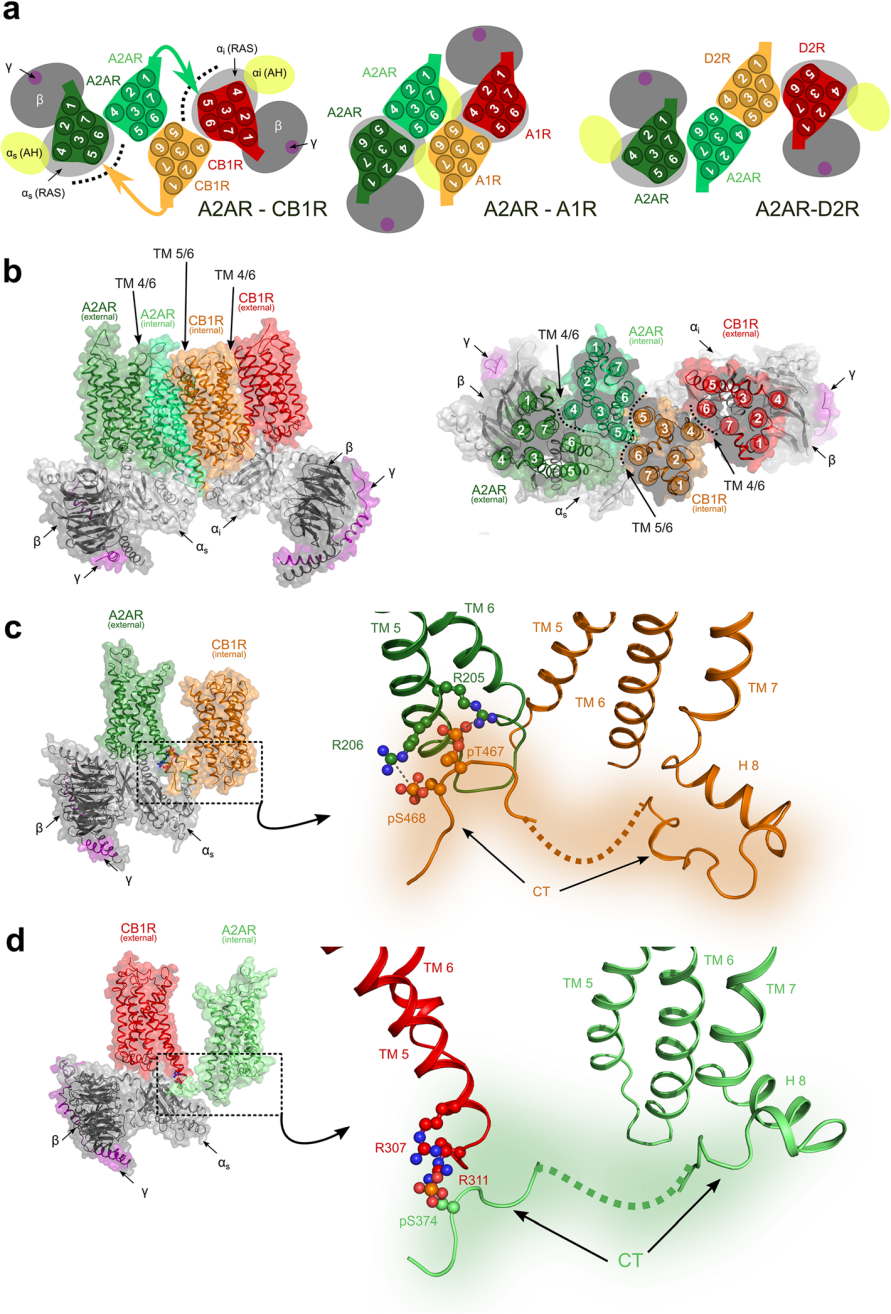
Quaternary structure of the A2AR-CB1R heterotetramer. **a** Schematic representation of the A2AR-CB1R heterotetramer (left) viewed from the extracellular side (colored arrows indicate the C-terminal segments of the internal protomers able to reach the external G protein-bound protomers) and the previously reported A2AR-A1R (middle) and A2AR-D2R (right) heterotetramers. Inactive/internal and active/external protomers of A2AR are shown in light and dark green, respectively, and inactive/internal and active/external protomers of CB1R, A1R, or D2R are shown in orange and red, respectively. The α-subunit of the G protein is shown in light gray, the β-subunit in dark gray, and the γ-subunit in purple. The α-helical domain (αAH) of the α-subunit, which performs a large-scale opening from the Ras domain, is shown in yellow. **b** A representative molecular representation of the A2AR-CB1R heterotetramer obtained from the MD simulation viewed from the membrane (left) or from the extracellular side (right). Color codes are as in panel **a**. **c** Proposed interaction between phosphorylated residues at the C-terminus of the CB1R (Gi-bound external protomer) with arginine residues at the end of TM 5 of the A2AR (internal protomer). **d** Proposed interaction between phosphorylated residues at the C-terminus of the A2AR (Gs-bound external protomer) with arginine residues at the end of TM 5 of the CB1R (internal protomer)

These results therefore demonstrate a change in a GPCR homodimeric interface imposed by an additional molecular interaction with another GPCR. Clearly, the observed TM 4/6 interface for CB1R-CB1R and A2AR-A2AR homodimerization in the A2AR-CB1R heterotetramer differs from the TM 4/5 interface for A1R-A1R and A2AR-A2AR homodimerization in the A1R-A2AR heterotetramer [6] and from the TM 6 interface for A2AR-A2AR and D2R-D2R homodimerization in the D2R-A2AR heterotetramer [7] (Fig. 3a). The almost complete reduction of fluorescence to background fluorescence levels induced by the TM6 peptide of A2AR in BiFC experiments with A2AR-nYFP and A2AR-cYFP indicate that, without the presence of A1R or CB1R, homodimerization with a TM 6 interface is the preferred oligomerization state of the A2AR, while higher-order A2AR oligomerization (homotrimers, heterotrimers) is clearly unlikely. Even though concomitant homomerization of A2AR by TM 6 or TM 4/5 interfaces is theoretically possible, our present and previous results indicate that the latter interface is conditional upon heteromerization with A1R.

We next constructed, using computational tools (see “Methods”), a structural model of the A2AR-CB1R heterotetramer using the information of the TM interfaces, for homo- (TM 4/6) and hetero- (TM 5/6) dimerization, derived from the BiFC experiments in the absence and presence of the TAT-fused peptides. The symmetrical TM 5/6 interface for heteromerization was modeled as in the crystal structure of the μ-opioid receptor [39]. The modeling of a symmetrical TM 4/6 interface for homomerization is not straightforward as it is not sterically feasible that these two helices in one protomer simultaneously interact with the same helices in the other protomer. To fit with the experimental data, in each homodimer, TM 4 of the internal protomer engaged in heteromerization should interact with TM 6 of the external protomer (an asymmetrical interface; Fig. 3a). In fact, crystal structures and molecular dynamics (MD) simulations have already provided evidence for the possibility that GPCRs show several possible symmetrical and asymmetrical homomeric TM interfaces [40, 41]. What the present results indicate is that the preferred homomeric interfaces can be determined by their heteromeric partner in a GPCR heterotetramer.

In our model of the A2AR-CB1R heterotetramer, Gi and Gs bind to the external protomer of the CB1R and A2AR homodimers, respectively, as we have previously suggested for the A1R-A2AR and A2AR-D2R heterotetramers [6, 7]. The fact that the C-terminal α5-helix of the G protein binds an intracellular cavity of the receptor that is opened mainly by the outward movement of TM 6 (TM 5 also moves but to a lesser extent), adds complexity to the modeling process. Thus, the TM 4/6 interface was modeled with the internal protomer in the inactive conformation (TM 6 closed) and the external protomer in the active conformation (TM 6 open). The stability of these interfaces was evaluated using MD simulations (see “Methods”), which yielded a converged structure for the complex with steady values for both root mean squared deviations and distance between protomers (not shown). Figure 3 shows a scheme (Fig. 3a) and two different views of the computational model (Fig. 3b). The mechanism for receptor catalyzed nucleotide exchange in G proteins involves a large-scale opening of the α-helical domain (αAH) of the α-subunit, from the Ras domain, allowing GDP to freely dissociate [42, 43]. Notably, the opening of αiAH and αsAH domains in the proposed models of the A2AR-CB1R and A2AR-D2R heteromers are pointing towards different directions (Fig. 3a [7];), while they are facing each other in the A1R-A2AR heteromer (Fig. 3b [6];).

### The C-terminal domain of the partner receptor determines the homomeric interfaces in the A2AR-CB1R heterotetramer

**Fig. 4:**
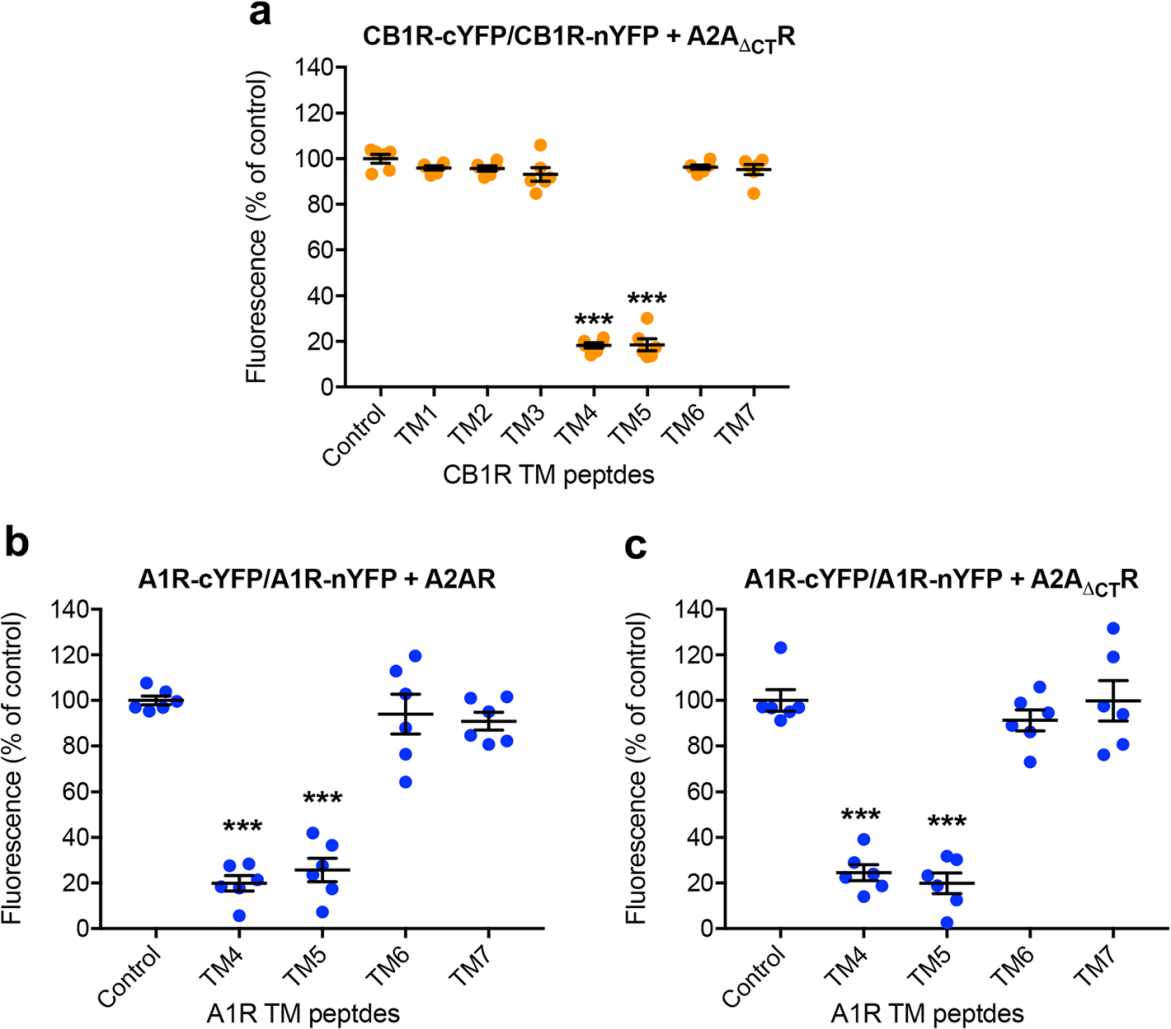
A2AR C-terminus-guided homomeric interface of the CB1R homodimer in the A2AR-CB1R heterotetramer. **a–c** BiFC experiments with synthetic peptides with the amino acid sequence of the TMs of CB1R and A1R. HEK-293T cells were transiently transfected with the following: **a** cDNA of two different molecules of CB1R separately fused to complementary halves of YFP (CB1R-cYFP and CB1R-nYFP; 3 μg in both cases) with co-transfection with cDNA of non-fused A2AR mutant lacking the C-terminal end (A2A_ΔCT_R; 1 μg); **b**, **c** cDNAs of two different molecules of A1R separately fused to complementary halves of YFP (A1R-cYFP and A1R-nYFP; 3 μg in both cases) with co-transfection with cDNA of non-fused A2AR (**b**; 1 μg) or non-fused A2A_ΔCT_R (**c**; 1 μg). Cells were treated for 4 h with medium (control) or 4 μM of indicated TM peptides (numbered 1–7). Values are means ± S.E.M. (*n* = 6 with triplicates in all the experiments) of the percentage of the fluorescence in the control group and analyzed statistically with repeated measures ANOVA, followed by Dunnett’s multiple comparison test (***: *p* < 0.001, compared with control)

A major question is the mechanism by which A2AR changes the CB1R-CB1R homomeric interface and CB1R changes the A2AR-A2AR interface (see above). A remarkable difference between the quaternary structure of the A2AR-CB1R heterotetramer and the A1R-A2AR and A2AR-D2R heterotetramers is the proximity of the C-terminal domain of the internal protomers to TM 5 and TM 6 of the external protomers (see arrows in Fig. 3a). Moreover, another difference among the three Gi-coupled partners of the A2AR is the long C-terminal domain of CB1R (73 residues) as compared to that of the A1R (34 residues) or the D2R (12 residues). We therefore speculated that the C-terminal domain of the partner receptor could be responsible for the change in the homomeric interface. To test this hypothesis, we engineered an A2AR mutant lacking the C-terminal end (A2A_ΔCT_R) and evaluated BiFC of CB1R-nYFP and CB1R-cYFP, in the presence and absence of each of the peptides of CB1R, and in the presence of A2A_ΔCT_R (Fig. 4a). While TM4 and TM6 reduced fluorescence in the presence of A2AR (Fig. 2f, see above), TM4 and TM5, but not TM6, reduced fluorescence in the presence of A2A_ΔCT_R (Fig. 4a). As an additional control, we tested whether A2A_ΔCT_R could modify the interface of the A1R homodimer in the A1R-A2AR heterotetramer. Clearly, the C-terminal domain of A2AR had no influence in the A1R homodimer (Fig. 4b, c). The same as for Fig. 2, the results in Fig. 4 are expressed as means ± S.E.M. of the percentage of the fluorescence in the control group, where control values (without interfering peptides) were always between 12,000 and 15,000 RFU. Non-transformed data show no significant change in fluorescence units upon co-transfection with a non-fused receptor. Thus, we can conclude that the C-terminal domain of A2AR modifies the CB1R-CB1R homomeric interface stabilizing the asymmetric TM 4/6 interface. Since the structure of the heterotetramer is symmetrical, we hypothesize that the C-terminal domain of CB1R also modifies the A2AR-A2AR homomeric interface stabilizing the asymmetric TM 4/6 interface. These conclusions are in agreement with previous results in which we described strong electrostatic interactions between phosphorylated Thr^467^-Ser^468^ at the C-terminal domain of CB1R and Arg^205^(5.66)-Arg^206^(5.67) at the intracellular end of TM 5 of A2AR as well as between phosphorylated Ser^374^ at the C-terminal domain of A2AR and the ^215^(5.64)VLRRRRKRVN^224^ epitope at the intracellular end of TM 5 of D2R [11]. Here, we propose similar interactions for the A2AR-CB1R heterotetramer. Although it is not possible to model the full-length C-terminal domain of either A2AR or CB1R, we modeled phosphorylated Ser^374^ of A2AR and phosphorylated Thr^467^-Ser^468^ of CB1R, connected by an arbitrary C-terminal domain (dotted lines in Fig. 3c, d). Figure 3c shows the electrostatic interactions between phosphorylated Thr^467^-Ser^468^ of CB1R and Arg^205^(5.66)-Arg^206^(5.67) of A2AR, and Fig. 3d shows the interaction between phosphorylated Ser^374^ of A2AR and Lys^300^(5.66)- Arg^307^(5.73)-Arg^311^(5.77) of CB1R. We hypothesize that these electrostatic interactions are the driving force for the conformational change, from the TM 4 interface of the CB1R homodimer in the absence of A2AR and from the TM 6 interface of the A2AR homodimer in the absence of CB1R, to the TM 4/6 interface that orients TM 5 towards the C-terminal domain of the partner receptor in the A2AR-CB1R heterotetramer.

### The A2AR-CB1R heterotetramer provides a frame for the canonical Gs-Gi antagonistic interaction at the AC level

**Fig. 5:**
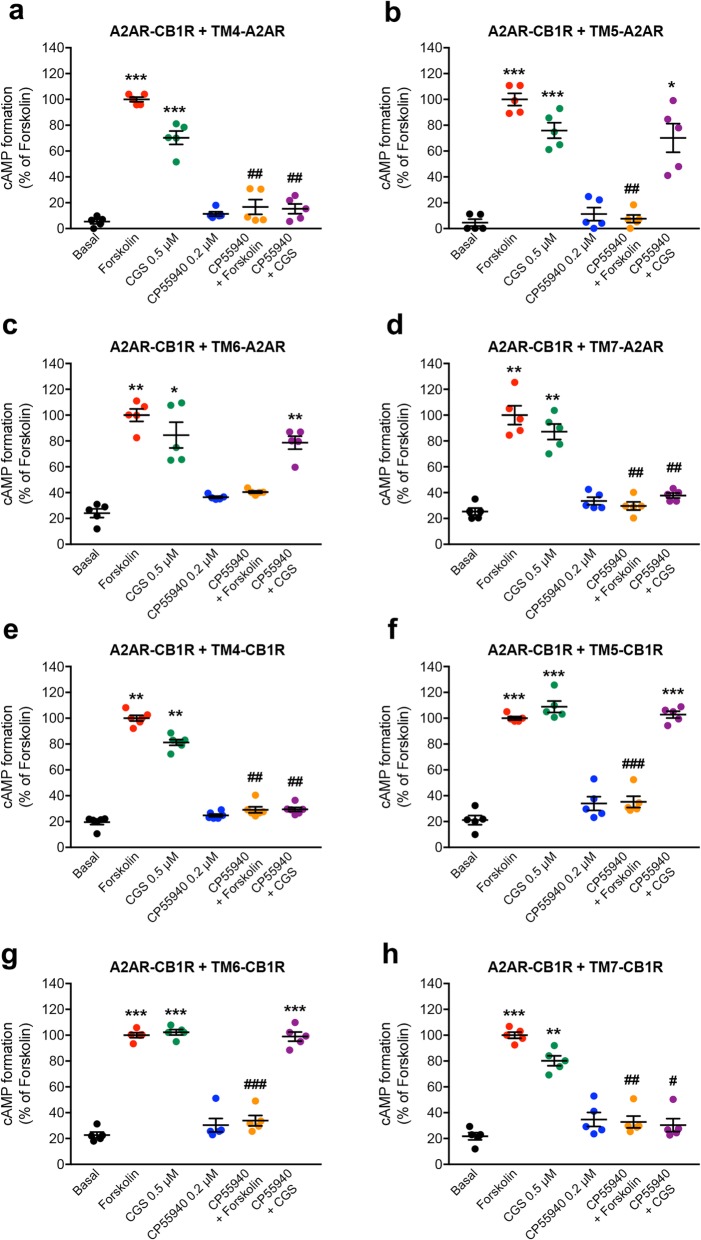
Canonical Gs-Gi antagonistic interaction at the AC level in the A2AR-CB1R heterotetramer. **a**–**h** Effect of TM peptides on the cAMP formation induced by forskolin (500 nM) or by the A2AR agonist CGS21680 (CGS, 500 nM) and counteractive effects of the CB1R agonist CP55940 (200 nM). HEK-293T cells were transiently transfected with the cDNAs of CB1R and A2AR (2 μg in both cases) and treated for 4 h with 4 μM of peptides TM4, TM5, TM6, or TM7 of the A2AR or CB1R. Values are means ± S.E.M. (*n* = 5 with triplicates in all experiments) of the percentage of forskolin-induced cAMP formation and analyzed statistically with repeated measures ANOVA, followed by Dunnett’s multiple comparison test (*, **, and ***: *p* < 0.05, *p* < 0.01, and *p* < 0.001, respectively, compared with basal; ^**#**^, ^**##**^, and ^**###**^: *p* < 0.01 and *p* < 0.001, respectively, compared to forskolin or CGS)

The ability of A2AR-CB1R heterotetramer to modulate AC signaling was then tested in HEK-293T cells by using heteromer-interfering peptides. As expected from their respective coupling to Gi and Gs proteins, the CB1R agonist CP55940 (200 nM) decreased cAMP formation induced by forskolin (500 nM) and the selective A2AR agonist CGS21680 (500 nM) increased cAMP in cells transfected with CB1R or A2AR, respectively (Additional file 1: Figure S4a and S4b). The same effect of both ligands was observed in cells co-transfected with A2AR and CB1R, where, in addition, CP55940 counteracted CGS21680-mediated increase in cAMP (Additional file 1: Figure S4c). This is in agreement with the ability of A2AR and CB1R to signal with their cognate G proteins and to establish the canonical Gs-Gi antagonistic interaction at the AC level in the A2AR-CB1R heterotetramer. Interfering peptides (TM4 to TM6 of CB1R and A2AR) were then used to demonstrate the dependence on the integrity of the A2AR-CB1R heterotetramer for the existence of this canonical Gs-Gi antagonistic interaction, as previously shown for the A2AR-D2R heterotetramer [7]. None of these peptides significantly modified the effect of either CP55940 or CGS21680 when activating their respective receptor (Fig. 5a–h; Additional file 2: Data values 3). On the other hand, TM5 and TM6 of both A2AR and CB1R, but not TM4 or TM7 (negative control), counteracted the canonical interaction (Fig. 5a–h; Additional file 2: Data values 3). Since TM 4 and TM 5 are selectively involved in homo- and heteromerization, respectively, while TM 6 is involved in both homo- and heteromerization, these results demonstrate that A2AR-CB1R heteromerization provides the necessary frame for the canonical Gs-Gi antagonistic interaction at the AC level. This effect was also present in the A2AR-D2R heterotetramer [7] but absent in the A1R-A2AR heterotetramer, which was devoid of canonical interaction [6]. This is rationalized by the fact that simultaneous opening of αiAH and αsAH is feasible in the A2AR-CB1R and A2AR-D2R heterotetramers (αAH opening is towards different direction) and is not feasible in A1R-A2AR because αiAH and αsAH face each other (Fig. 3a) [6].

Experiments were also performed in cells transfected with both receptors and pre-treated with pertussis toxin (PTX) or cholera toxin (CTX) (for details, see legend to Additional file 1: Figure S4), which alter Gi- and Gs-mediated signaling, respectively. As expected, we observed blockade of CP55940-induced cAMP decrease by PTX, and blockade of CGS21680-induced cAMP increase by CTX (Additional file 1: Figure S4d and S4e). In addition, PTX and CTX also blocked the effect of CGS21680 and CP55940, respectively (Additional file 1: Figure S4d and S4e). These results indicate that both A2AR- and CB1R-mediated signaling in the A2AR-CB1R heterotetramer are dependent on the functional integrity of both Gs and Gi proteins. The same phenomenon has been previously described for the A1R-A2AR heteromer [6, 44], while the toxins maintained their expected selectivity for either Gs or Gi in the A2AR-D2R heteromer [7]. This cross-communication is not fully understood but it could be related to the closer proximity of the α-subunits of Gs and Gi coupled to the A2AR-CB1R and A1R-A2AR heterotetramers.

### Presynaptic A2AR-CB1R heterotetramers mediate the cannabinoid-induced inhibition of striatal glutamate release

**Fig. 6:**
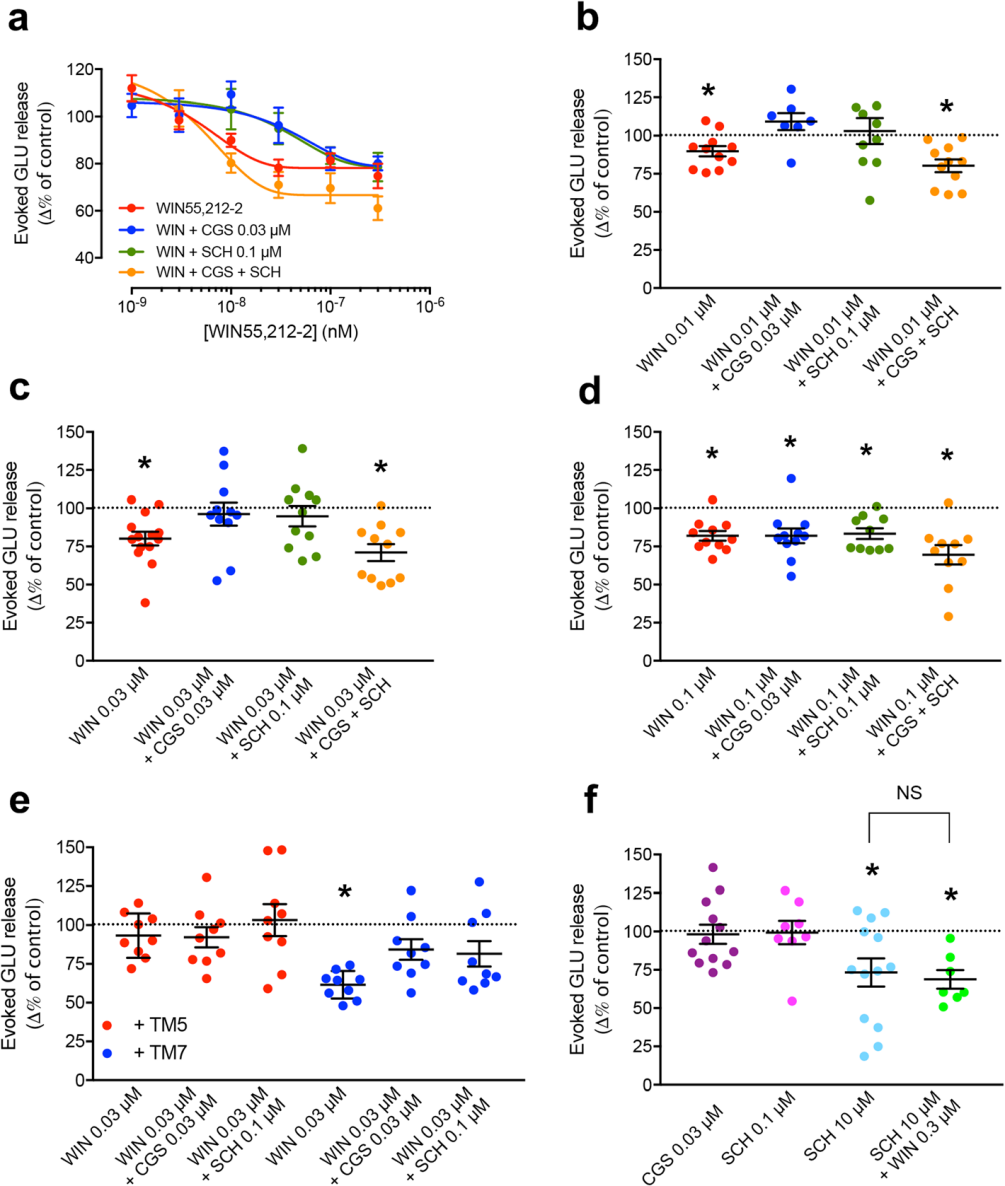
A2AR-CB1R heteromer-mediated control of the evoked glutamate release from striatal nerve terminals. **a** Concentration-response curves of the inhibitory effect of WIN55,2121-2 (WIN) on the evoked glutamate release, expressed as the difference in % of the control value, alone or in the presence the A2AR agonist CGS21680 (CGS, 0.03 μM), the A2AR antagonist SCH58261 (SCH, 0.1 μM), or both. Data are means ± S.E.M. of 7–14 experiments with duplicates per treatment and concentration. **b**–**d** Individual results from the experiments shown in **a** with WIN at the concentrations of 0.01 μM (**b**), 0.03 μM (**c**), and 0.1 μM (**d**), administered alone or in the presence of CGS (0.03 μM), SCH (0.1 μM), or both (yellow dots). **e** Effect of the peptides TM5 and TM7 of the A2AR on the inhibitory effect of WIN (0.03 μM) on the evoked glutamate release, expressed as the difference in % of the control value, alone or in the presence of CGS (0.03 μM) or SCH (0.1 μM). **f** Effect of CGS (0.03 μM), SCH (0.1 and 10 μM), or SCH (10 μM) plus WIN (0.3 μM). **b**–**f** Values are means ± S.E.M. (*n* = 7–14 with duplicates) of the evoked glutamate release, expressed as the difference in % of the control value, and analyzed statistically with one-sample *t*-test (*: *p* < 0.05/3; see “Methods”)

The demonstration of the presence and functional significance of A2AR-CB1R heteromers in striatal glutamatergic terminals was then approached by analyzing the ability of the CB1R agonist WIN55,212-2 to inhibit depolarization-induced glutamate release from rat striatal synaptosomes (see “Methods” and ref. [19]). We would first expect that, as previously reported [19, 21], A2AR agonists would reduce the effect, and more specifically the potency, of WIN55,212-2. Second, we should be able to demonstrate that A2AR antagonists would produce the same effect as A2AR agonists and that when co-applied they would counteract each other’s effect. Third, if these effects of A2AR agonists and antagonists would reflect functional correlations of the allosteric interactions within the A2AR-CB1R heterotetramer, they should be counteracted by a synthetic peptide that destabilizes the heteromeric interface. Finally, we should also find a correlate of the Gs-Gi canonical interaction at the AC level. Indeed, as shown in Fig. 6a–d, WIN55,212-2 inhibited evoked glutamate release (EC_50_, 5.5 nM) in a concentration-dependent fashion, reaching its maximal effect (77.0 ± 3.0% of DMSO control) at 0.03 μM. The concentration-response inhibitory curve of WIN55,212-2 was shifted to the right both by the A2AR agonist CGS21680 (EC_50_, 36.8 nM) and the A2AR antagonist SCH58261 (EC_50_, 40.0 nM) alone, but not when co-administered (EC_50_, 8.2 nM). In detail, CGS21680 (0.03 μM) and SCH58261 (0.1 μM) fully prevented the inhibition by 0.01 and 0.03 μM, but not by 0.1 μM of WIN55,212-2 of glutamate release (Fig. 6a–d). On the other hand, co-application of both CGS21680 (0.03 μM) and SCH58261 (0.1 μM) did not significantly modify the effect of WIN55,212-2 (Fig. 6a–d).

Striking results were obtained when evaluating the effect of the destabilizing A2AR-CB1R heteromer peptides. TM5, but not TM7, of A2AR significantly blocked WIN55,212-2-induced inhibition of glutamate release and no additional change was observed with the application of the A2AR ligands (Fig. 6e). These results would indicate that WIN55,212-2-induced inhibition of glutamate release is dependent on A2AR-CB1R heteromerization and would confirm the initial hypothesis of the permissive role of A2AR for CB1R signaling in the A2AR-CB1R heteromer [18]. But the results would also indicate that the permissive role of A2AR would not rely on a ligand-dependent A2AR activation.

Our assumption about presynaptic A2AR-CB1R heteromers determining all interactions between A2AR and CB1R ligands at the level of striatal glutamatergic transmission bears the question of how the activation of A2AR can be needed for CB1R signaling while, at the same time, A2AR ligands significantly decrease the potency of CB1R ligands. A possible answer to this apparent conundrum is the existence of a significant constitutive activity of A2AR in the A2AR-CB1R heteromer, which would produce a constitutive activation of AC. A CB1R receptor ligand, including endocannabinoids, would inhibit A2AR-mediated constitutive AC activation by the canonical Gi-Gs antagonistic interaction in the absence of A2AR ligands. In fact, a high constitutive activity of A2AR has been previously reported [45–47]. This would first imply that the CB1R-mediated inhibition of glutamate release is dependent on a Gi-coupling with or without heteromerization with A2AR. Synaptosomes were incubated for 4 h at 4 °C with or without PTX (2 μg/ml) before the regular release experiment procedure. While WIN55,212-2 (0.1 μM) significantly inhibited glutamate release in the toxin-naïve synaptosomes (in mean ± S.E.M and expressed as the difference in % of the control value: 82.9% ± 3.6%, *n* = 6; one-sample *t*-test: *p* < 0.05/3; see “Methods”), PTX fully prevented this inhibition (in mean ± S.E.M and expressed as the difference in % of the control value: 114.6% ± 6.8, *n* = 6; one-sample *t*-test: *p* > 0.05/3). Finally, one would also expect that a high concentration of the A2AR inverse agonist, SCH58261 [46], should lead to a significant reduction of the ability of WIN55,212-2 to inhibit evoked glutamate release. Indeed, at 10 μM (but not 0.1 μM), SCH58261 significantly inhibited the evoked glutamate release, which could not be further inhibited by a relatively high concentration of WIN55212-2 (0.3 μM; Fig. 6f). These results therefore indicate that a main mechanism by which CB1R localized in striatal nerve terminals modulate glutamate release is by counteracting a constitutive A2AR-mediated facilitation of glutamate release by a canonical Gi-Gs antagonistic interaction dependent on A2AR-CB1R heteromerization.

**Fig. 7:**
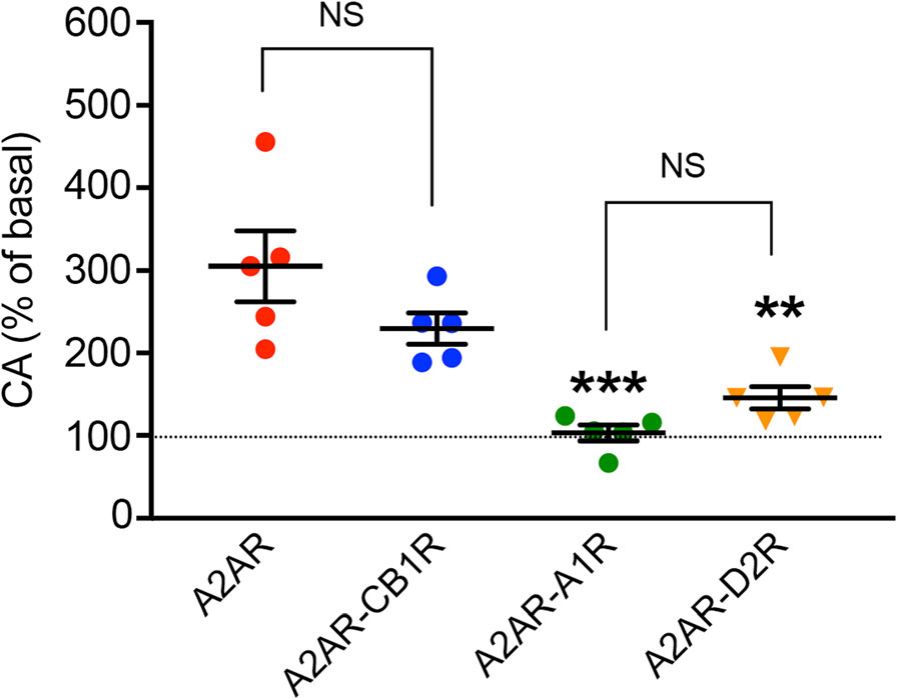
Differential A2AR constitutive activity (CA) in different A2AR heteromers. Constitutive activation of AC in cells expressing A2AR alone or with A2AR plus CB1R, A1R, or D2R. HEK-293T cells were transiently transfected with the cDNA of A2AR alone (3 μg) or with the cDNA of A2AR (1.5 μg) and the cDNA of CB1R, A1R, or D2R (1.5 μg in all cases). CA values are expressed as means ± S.E.M. (*n* = 5 with quintuplicates in all experiments) of the % of basal values, calculated by dividing the levels of cAMP obtained in each condition by the levels of cAMP in cells transfected with an empty vector (pcDNA3, 3 μg). In addition, the values were normalized by the levels of cell surface expression of A2AR, which was determined by means of SNAP staining (see “Methods”). Statistical analysis was performed by using one-way ANOVA, followed by Tukey’s multiple comparison test (** and ***: *p* < 0.01 and *p* < 0.001, respectively, compared with cells only transfected with A2AR)

The existence of a significant constitutive activity of A2AR in the A2AR-CB1R heterotetramer was confirmed with experiments in HEK-293T cells transiently transfected with A2AR alone or with additional transfection with CB1R, A1R, or D2R. A2AR showed a high constitutive activity (about 250% increase in cAMP formation in cells only transfected with A2AR, as compared with cells transfected with an empty control vector; see “Methods”) (Fig. 7; Additional file 2: Data values 4). Very dramatic differential results were obtained when A2AR was co-transfected with either CB1R, A1R, or D2R. Thus, A2AR lost its constitutive activity when co-transfected with A1R or D2R, but it was not significantly modified with co-transfection with CB1R (Fig. 7; Additional file 2: Data values 4). Therefore, these results indicate that the constitutive activity of A2AR is blunted by heteromerization with either A1R or D2R, but not by heteromerization with CB1R; activation of CB1R in the A2AR-CB1R heterotetramer can, nevertheless, blunt the A2AR-mediated constitutive activation of AC (see “Discussion”). The molecular mechanism of this heteromerization-dependent control of the constitutive activation of A2AR seems difficult to rationalize, but we hypothesize that the postulated interaction between TM 5 of the Gs-bound A2AR protomer and the C-terminal domain of the Gi-unbound CB1R protomer (Fig. 3c), may help to sustain the constitutive activity of A2AR in the A2AR-CB1R heterotetramer, in contrast to the short C-termini of the A1R or D2R in the A1R-A2AR and A2AR-D2R heterotetramers (Fig. 3a). In fact, TM 5 and TM 6 are the helices that move upon receptor activation.

## Discussion

In the present study, using a peptide-interfering approach combined with biophysical and biochemical techniques in mammalian transfected cells and computational modeling, we could establish the tetrameric quaternary structure of the A2AR-CB1R heterotetramer, having the same heteromeric but different homomeric interfaces than those of the A1R-A2AR heterotetramer. The long A2AR-CT played a significant role, determining the homomeric interface of the CB1R homodimer. The A2AR-CB1R heterotetramer showed the same allosteric interactions and canonical Gs-Gi antagonistic interaction at the AC level as the A2AR-D2R heterotetramer. The utilization of TAT-fused TM peptides in mammalian transfected cells and striatal primary neuronal cultures allowed demonstrating that the canonical interaction at the AC level between A2AR and D2R ligands required the right quaternary structure of the A2AR-D2R heterotetramer in the A2AR-D2R-AC5 complex. Application of TM peptides that specifically interfere with the heteromeric interface or with the receptor-AC5 interface specifically counteracted the ability of a D2R agonist to inhibit AC5 activation by an A2AR agonist, but not with the ability of D2R ligands to counteract forskolin-induced AC activation [7]. More generally, these results indicate that to inhibit a Gs-coupled receptor-mediated AC activation, the ligands need to simultaneously interact with heterotetramers of the corresponding Gs-coupled and Gi-coupled receptors. This has been so far shown for the A2AR-D2R [7], the A1R-D1R [48], the D1R-D3R [49], and now the A2AR-CB1R heterotetramers, where destabilization of their heteromeric interface leads to the disruption of the canonical Gs-Gi antagonistic interaction at the AC level without disruption of the Gi-coupled receptor-mediated inhibition of forskolin-induced AC activation.

Different from the A2AR-D2R and A1R-A2AR heterotetramers, the A2AR-CB1R heterotetramer showed a significant A2AR-mediated constitutive activation of AC. Using the peptide-interfering approach in rat striatal synaptosomal preparations, we could then identify the A2AR-CB1R heteromer in striatal glutamatergic terminals, where it provides the main target by which cannabinoids inhibit glutamate release. The present study presents a new conceptual view about the mechanism by which cannabinoids presynaptically control striatal excitatory neurotransmission. Instead of the classically assumed direct inhibition of glutamate release by CB1R-mediated signaling, we demonstrate that cannabinoids inhibit glutamate release indirectly, by counteracting the stimulation of glutamate release induced by A2AR-mediated signaling. This mechanism is completely dependent on the heteromerization with A2AR, which provides the frame for the canonical Gi-Gs antagonistic interaction at the AC level. In addition, this mechanism operates in the absence of an adenosine-mediated activation of the A2AR, by the ability of CB1R activation to cancel A2AR-mediated constitutive activation of AC within the A2AR-CB1R heteromer. We could also demonstrate that the constitutive activity is also present when the A2AR is not forming heteromers and that it is lost upon heteromerization with A1R or D2R.

We recently provided the proof of concept of a ligand-independent ability of one of the protomers in a GPCR heteromer to modify the constitutive activity of the other molecularly different protomer. That was the ability of the dopamine D4 receptor (D4R) to modify the constitutive activity in the D2R-D4R heteromer [32], also localized in striatal glutamatergic terminals, where it mediates a presynaptic dopaminergic inhibitory control of glutamate release [50–52]. This effect was even dependent on the polymorphic variant of the D4R, demonstrating for the first time a functional difference between the two most common D4R polymorphic variants, D4.4 and D4.7 [52]. The D4.7R-mediated increase in the constitutive activity of the D2R could explain the recently reported gain of function of the D2R in the control of striatal glutamate release when forming heteromers with the ADHD-associated polymorphic variant D4.7R [52]. The constitutive activity of the D2R in the D2R-D4R heteromers and the constitutive activity of the A2AR in the A2AR-CB1R seem therefore to determine the basal degree of sensitivity of striatal glutamatergic terminals to release glutamate upon depolarization.

The two A2AR heteromers, A2AR-CB1R and A1R-A2AR, allow an adenosine-mediated fine-tuned modulation of presynaptic striatal glutamatergic transmission, which is moderated by the extracellular levels of endocannabinoids [2] (Additional file 1: Figure S5). First, the A2AR loses its constitutive activity when it forms heteromers with A1R and adenosine binds with more affinity to A1R than to A2AR. As mentioned in the “Background”, low extracellular concentrations of adenosine should predominantly activate A1R within the A1R-A2AR heteromer, leading to a predominant inhibition of glutamate release (Additional file 1: Figure S5a). This would represent a mechanism of silencing corticostriatal “noise”. Retrograde endocannabinoid release following postsynaptic metabotropic glutamate receptor activation [25] would further guarantee the low noise, by counteracting the A2AR-mediated constitutive activation of AC by the A2AR-CB1R heteromer (Additional file 1: Figure S5a). Upon strong corticostriatal activity, the increase in the presynaptic release and degradation of ATP would create locally high levels of adenosine, which in turn would activate A2AR to shut down A1R signaling in the A1R-A2AR heteromer [3, 6] and to counteract CB1R signaling in the A2AR-CB1R heteromer, switching the effect of adenosine on glutamate release from inhibition to facilitation (Additional file 1: Figure S5b). This high-pass filter is expected to increase the reliability of information passage and should be maximized under conditions of low extracellular concentrations of endocannabinoids [2].

The present study seems to be in contradiction with our recent previous study, where A2AR-CB1R heteromers were described to be preferentially coupled to Gq and postsynaptically localized in the striatum, in GABAergic striatopallidal neurons [53], while the present study shows their presynaptic localization in glutamatergic terminals and their preferential coupling to Gs and Gi proteins. Although CB1R couples preferentially to Gi/o proteins, its ability to couple to Gq has already been reported and shown to be ligand-dependent [53]. Thus, WIN55,212-2 was found to be more and less potent than CP55940 at eliciting Gq-dependent intracellular calcium increase and at inhibiting forskolin-induced cAMP production, respectively [53, 54]. Nevertheless, despite its relative functional selectivity for Gq protein-mediated signaling, in the present study, WIN55,212-2 was shown to inhibit striatal glutamate release by a PTX-sensitive mechanism. Therefore, the results of our previous and present studies indicate that the preference for a specific G protein coupling of the A2AR-CB1R heteromer depends mostly on the cellular environment. They also suggest that Gq coupling depends on an additional component that is present in immortalized striatal neuroblasts and GABAergic striatopallidal neurons (previous study) and absent in HEK-293T cells and glutamatergic terminals (present study). One very plausible candidate is the canonical Gq-coupled glutamate mGlu5 metabotropic receptor, which is abundantly expressed postsynapytically in the striatum [25]. Thus, mGlu5R was previously reported to form functional heteromers with A2AR [55, 56] and suggested to form complexes that include the three different GPCRs [57]. Experiments are in progress to determine if the striatal Gq protein coupling of postsynaptic A2AR and CB1R depends on their ability to separately or simultaneously form heteromers with mGlu5R.

The important role of the A2AR-CB1R heteromer in the inhibitory control of corticostriatal glutamate release makes it a potential target for neuropsychiatric disorders putatively associated with increased corticostriatal transmission, which includes obsessive-compulsive disorder [58], schizophrenia [59], and substance use disorders [60]. In addition, changes in the stoichiometry of the presynaptic A1R-A2AR and A2A-CB1R receptor heteromers could have important implications in the pathogenesis of neuropsychiatric disorders. For instance, in Restless Legs Syndrome (RLS), there is recent preclinical evidence for the existence of downregulation of striatal A1R [61], which promotes an increased sensitivity of corticostriatal terminals to release glutamate [51, 62]. This should be associated with a relative functional upregulation of presynaptic A2AR due to an increase in the proportion of A2AR not forming heteromers or forming heteromers with CB1R, both endowed with constitutive activity. As expected, the increased sensitivity of corticostriatal terminals observed in the experimental animal could be counteracted by blocking the constitutive signaling of A2AR, with A2AR inverse agonists or CB1R agonists, or by activating A1R, by moderately increasing extracellular levels of adenosine with an inhibitor of the adenosine transporter. There is in fact preliminary clinical evidence for the successful application of these strategies in RLS patients [63–65].

Finally, more studies need to be done to evaluate the possible existence of A2AR-CB1R heteromers, and therefore, the same type of adenosine-cannabinoid-mediated modulation of glutamate release, in other areas of the CNS. It is very plausible that A2AR-CB1R heteromers modulate neurotransmitter release in many other brain areas, such as the hippocampus. Those heteromers could mediate the reported ability of A2AR antagonists to prevent cannabinoid-induced memory and LTP impairments [66, 67].

## Conclusions

We demonstrate that the ability of cannabinoids to control excitatory transmission in the striatum depends on adenosine transmission, which is mediated by heteromers of specific subtypes of adenosine and cannabinoid receptors, A2AR and CB1R. The molecular analysis of the A2AR-CB1R heteromers supports our hypothesis about GPCR heteromers being often constituted by heteromers of homodimers [68]. It also supports that these GPCR heterotetramers often include Gs- and Gi-coupled homodimers, providing the frame for the canonical Gs-Gi antagonistic interaction at the adenylyl cyclase level, forming part of GPCR signaling complexes that include GPCR heteromers and their common interacting effectors [7, 69]. The comparison of the A2AR-CB1 receptor with other heterotetramers of A2AR added new information about the properties of GPCRs. It revealed that different heteromeric partners of A2AR determine profound differences in their pharmacological properties. This implies the need to consider the immediate context of a GPCR to truly understand its pharmacological properties and, therefore, its role in drug development.

## Methods

### Expression vectors and fusion proteins

Sequences encoding amino acids (aa) residues 1-229 and 230-311 of Rluc (Rluc8 variant) and amino acid residues 1-155 and 156-238 of YFP (mVenus variant) were subcloned in pcDNA3.1 vector to obtain complementary Rluc and YFP hemi-truncated proteins (nRluc, cRluc, nYFP, and cYFP). The cDNAs for human CB1R, A2AR, A1R, D1R, D2R (short isoform), and 5-HT2AR cloned into pcDNA3.1 were amplified without their stop codons using sense and antisense primers harboring EcoRI and BamHI sites to clone A2AR and D1R or EcoRI and KpnI to clone A1R and CB1R. The amplified fragments were subcloned to be in-frame with restriction sites of pcDNA3.1-nYFP, pcDNA3.1-cYFP, pcDNA3.1-nRluc, or pcDNA3.1-Rluc vectors to provide plasmids that express the receptor fused to nYFP, cYFP, nRluc, or cRluc on the C-terminal end of the receptor. The following human G protein constructs were used: Gαi1-YFP (with YFP, mVenus variant, inserted at position 91), Gαs-YFP (with YFP, mVenus variant, inserted at position 154 of Gαs, short isoform), and Gαq-YFP (with YFP, mVenus variant, inserted at position 97 of Gαs), untagged Gβ1, and untagged Gγ2. All the constructs were confirmed by sequencing analysis. Several constructs were shared by C. Gales at INSERM (Toulouse, France; Gαi1 construct) and N. Lambert (Georgia Regents University, Augusta, GA; Gαs). The expression of constructs was tested by confocal microscopy and the receptor-fusion protein functionality by ERK1/2 phosphorylation. An A2AR mutant with a deletion of aa 321 to aa 412 on the C-terminal domain of A2AR (A2A_ΔCT_R) was generated as previously described [70].

### HIV TAT-fused TM peptides

Peptides with the amino acid sequence of TMs of the CB1R, A2AR, and A1R were used as oligomer-destabilizing agents, as previously demonstrated [6, 7, 12, 31, 71]. To allow intracellular delivery, a peptide can be fused to the cell-penetrating HIV transactivator of transcription (TAT) peptide (YGRKKRRQRRR). HIV TAT fused to a TM GPCR peptide can be inserted effectively into the plasma membrane as a result of both the penetration capacity of the TAT peptide and the hydrophobic property of the TM peptide [72]. To obtain the right orientation of the membrane-inserted peptide, HIV TAT peptide was fused to the C-terminus of peptides with the amino acid sequence of TM 1, TM 3, TM 5, and TM 7 of CB1R, A2AR, or A1R (TM1, TM3, TM5, and TM7 peptides, respectively) or to the N-terminus of TM 2, TM 4, and TM 6 of CB1R, A2AR, or A1R (TM2, TM4, and TM6 peptides, respectively). All peptides were synthesized by Genemed Synthesis, Inc. Their sequences were as follows:VYITVELAIAVLAILGNVLVCWAVWYGRKKRRQRRR for TM1 of A2AR,YGRKKRRQRRRYFVVSLAAADIAVGVLAIPFAITI for TM2 of A2AR,LFIACFVLVLTQSSIFSLLAIAIYGRKKRRQRRR for TM3 of A2AR,YGRKKRRQRRRAKGIIAICWVLSFAIGLTPMLGW for TM4 of A2AR,MNYMVYFNFFACVLVPLLLMLGVYLYGRKKRRQRR R for TM5 of A2AR,YGRKKRRQRRRLAIIVGLFALCWLPLHIINCFTFF for TM6 of A2AR,LWLMYLAIVLSHTNSVVNPFIYAYYGRKKRRQRRR for TM7 of A2AR.LAIAVLSLTLGTFTVLENLLVLCVILYGRKKRRQRRR for TM1 of CB1R,YGRKKRRQRRRFIGSLAVADLLGSVIFVYSFI for TM2 of CB1R, FIGSLAVADLLGSVIFVYSFIYGRKKRRQRRR for TM3 of CB1R,YGRKKRRQRRRAVVAFCLMWTIAIVIAVLPLLGW for TM4 of CB1R,TYLMFWIGVTSVLLLFIVYAYMYILWYGRKKRRQRRR for TM5 of CB1R,YGRKKRRQRRRLVLILVVLIICWGPLLAIMVY for TM6 of CB1R,VFAFCSMLCLLNSTVNPIIYALYGRKKRRQRRR for TM7 of CB1RRRRQRRKKRGYAAVAIAGCWILSFVVGLTPMFGW for TM4 of A1R,MEYMVYFNFFVWVLPPLLLMVLIYLYGRKKRRQRRR for TM5 of A1R,RRRQRRKKRGYLALILFLFALSWLPLHILNCITLF for TM6 of A1R,ILTYIAIFLTHGNSAMNPIVYAFRIYGRKKRRQRRR for TM7 of A1R.

### Cell cultures and transient transfection

Human embryonic kidney 293T (HEK-293T) cells obtained from ATCC were grown in Dulbecco’s modified Eagle’s medium (DMEM) (Gibco, Gaithersburg, MD) supplemented with 2 mM l-glutamine, 100 μg/ml sodium pyruvate, 100 U/ml penicillin/streptomycin, MEM non-essential amino acid solution (1/100), and 5% (v/v) heat inactivated fetal bovine serum (FBS) (all supplements were from Invitrogen, Paisley, Scotland, UK). Cells were maintained at 37 °C in an atmosphere of 5% CO_2_. For transient transfection, HEK-293T cells growing in six-well dishes were transfected with the corresponding fusion protein cDNA by the PEI (PolyEthylenImine, Sigma-Aldrich, Saint Louis, MO) method. Cells were incubated (4 h) with the corresponding cDNA together with PEI (5.47 mM in nitrogen residues) and 150 mM NaCl in a serum-starved medium. After 4 h, the medium was changed to a fresh complete culture medium. Forty-eight hours after transfection, cells were washed twice in quick succession in Hank’s balanced salt solution (HBSS; containing, in mM: 137 NaCl, 5 KCl, 0.34 Na_2_HPO_4_, 0.44 KH_2_PO_4_, 1.26 CaCl_2_, 0.4 MgSO_4_, 0.5 MgCl_2_, 10 HEPES, pH 7.4), supplemented with 0.1% glucose (w/v), detached, and resuspended in the same buffer. To control the cell number, sample protein concentration was determined using a Bradford assay kit (Bio-Rad, Munich, Germany) using bovine serum albumin dilutions as standards.

### Bimolecular fluorescence complementation (BiFC)

HEK-293T cells transfected with the receptor fused to n-YFP and the receptor fused to the cYFP were treated with vehicle or the indicated TAT-fused TM peptides (4 μM) for 4 h at 37 °C. To quantify protein-reconstituted YFP Venus expression, cells (20 μg protein) were distributed in 96-well microplates (black plates with a transparent bottom; Porvair, King’s Lynn, UK), and emission fluorescence at 530 nm was read in a Fluo Star Optima Fluorimeter (BMG Labtechnologies, Offenburg, Germany) equipped with a high-energy xenon flash lamp, using a 10-nm-bandwidth excitation filter at 400 nm reading. Protein fluorescence was determined as fluorescence of the sample minus fluorescence of non-transfected cells. Cells expressing CB1R, A2AR, A1R, or D1R fused to nYFP or to cYFP, as well as cells expressing nYFP or cYFP or both complementary proteins (not fused to receptors) showed similar fluorescence levels to non-transfected cells.

### Bioluminescence resonance energy transfer (BRET) with donor and acceptor complementation

HEK-293T cells were transfected with the corresponding receptors fused to nYFP, cYFP, nRluc, and cRluc (see figure legends). To quantify receptor-YFP expression, cells (20 μg protein) were distributed in 96-well microplates (black plates with a transparent bottom) and fluorescence at 530 nm was read as described above. Receptor fluorescence was determined as fluorescence of the sample minus the fluorescence of cells expressing only the BRET donor. For BRET measurements, cells (20 μg protein) were distributed in 96-well microplates (Corning 3600, White plates; Sigma-Aldrich) and BRET signal was collected 1 min after addition of 5 μM coelenterazine H (Molecular Probes, Eugene, OR) using a Mithras LB 940 microplate reader (Berthold Technologies, Bad Wildbad, Germany), which allows integration of the signals detected in the short-wavelength filter at 485 nm (440–500 nm) and the long-wavelength filter at 530 nm (510–590 nm). To quantify receptor-Rluc reconstitution, luminescence readings were also performed after 10 min of adding 5 μM of coelenterazine H. Both the fluorescence and luminescence of each sample were measured before each experiment to confirm similar donor expression (∼150,000 luminescent units). Net BRET is defined as [(long-wavelength emission)/(short-wavelength emission)]-Cf where Cf corresponds to [(long-wavelength emission)/(short-wavelength emission)] for the Rluc construct expressed alone in the same experiment. BRET is expressed as milli BRET units (mBU; net BRET × 1000).

### Complemented donor-acceptor resonance energy transfer (CODA-RET)

HEK-293T cells transfected with A2AR fused to cRluc and CB1R fused to nRluc, or 5-HT2AR fused to cRluc and D2R (short isoform) fused to nRluc, and co-transfected with either Gαs, Gαi1, or Gαq fused to YFP were harvested, washed, and resuspended in phosphate-buffered saline (PBS). About 200,000 cells/well were distributed in 96-well plates, and 5 μM coelenterazine H was added to each well. One minute after addition of coelenterazine, different concentrations of the non-selective adenosine receptor agonist NECA, the selective CB1R agonist CP55940, dopamine, or serotonin were added to each well. In some experiments, NECA or caffeine were added 12 min before the addition of CP55940. Fluorescence of the acceptor was quantified (excitation at 500 nm and emission at 540 nm for 1-s recording) in the Mithras LB940 reader to confirm the constant expression level across experiments. In parallel, BRET signal from the same batch of cells was determined as the ratio of the light emitted by YFP (mVenus variant; 510–540 nm) over Rluc (485 nm). Results were calculated for the BRET change (BRET ratio for the corresponding drug minus BRET ratio in the absence of the drug) 10 min after addition of the agonists. Data manipulations and statistical analyses for all CODA-RET, BRET, and complementation experiments were performed with GraphPad Prism 7.0 software (La Jolla, CA).

### cAMP accumulation

cAMP accumulation was measured using the LANCE Ultra cAMP kit (PerkinElmer, Waltham, MA, USA) as previously described [73]. In brief, HEK-293T cells were seeded in white 384-wells plates in Dulbecco’s modified Eagle’s medium (DMEM) (Sigma-Aldrich) containing zardaverine (up to 50 μM; Calbiochem, San Diego, CA, USA) and, when using A2AR ligands, adenosine deaminase (ADA, 0.5 U/ml; Roche Diagnostics, GmbH, Mannheim, Germany). Cells were incubated for 1 h at 37 °C and for 4 h at 37 °C when using TAT-fused TM peptides. Subsequently, selective ligands and/or forskolin were added and cells were incubated for 30 min. Finally, Eu-cAMP tracer and ULight™-anti-cAMP reagents were prepared and added to the sample following the manufacturer’s instructions. The 384-wells plate was incubated 1 h at 22 °C in the dark and was then read on a CLARIOstar or PHERAstar microplate reader (BMG Labtech, Durham, NC, USA). Measurements at 620 nm and 665 nm were used to detect the TR-FRET signal and the concomitant cAMP levels were calculated following the manufacturer’s instructions. Data were fitted by non-linear regression using GraphPad Prism 6.01 (San Diego, CA, USA). When comparing the effect of different co-expression experiments, cells were labeled by means of SNAP staining as previously described [47] and cAMP values were normalized according to the levels of cell surface expression of A_2A_R in each condition (A2AR, A2AR + A1R, A2AR + CB1R, A2AR + D2R). In brief, adherent cells expressing the A_2A_R^SNAP^ were washed and incubated with DMEM containing 100 nM of SNAP-surface 488 substrate (New England BioLabs, Ipswich, MA) for 1 h at 37 °C. Cells were then washed three times with phenol red-free Hank’s balanced salt solution containing 1 g/l glucose (HBSS: 137 mM NaCl, 5.4 mM KCl, 0.3 mM Na_2_HPO_4_, 0.4 mM KH_2_PO_4_, 4.2 mM NaHCO_3_, 1.3 mM CaCl_2_, 0.5 mM MgCl_2_, 0.6 mM MgSO_4_, 5.6 mM glucose, pH 7.4) and fluorescence read on the CLARIOstar microplate reader (BMG Labtech).

### Computational model of the A2AR-CB1R heterotetramer

The structural model of the A2AR-CB1R heterotetramer consists of a heteromer of homodimers. The A2AR homodimer is formed by an internal inactive protomer (interacting with CB1R), modeled with PDB id 5NM4 [74, 75], and an external active protomer bound to Gs, modeled with PDB id 5G53 of A2AR in complex with a mini-Gs α-subunit [76, 77]. Gs was modeled based on the mini-Gs α-subunit and the βγ-subunits of the A2AR-Gs structure with PDB id 6GDG [78, 79]. The CB1R dimer is formed by an internal inactive protomer (interacting with A2AR), modeled with PDB id 5U09 [80, 81], and an external active protomer bound to Gi, modeled with PDB id 6N4B of the CB1R-Gi complex [82, 83]. The A2AR-CB1R heteromer was modeled via the TM 5/6 interface, using the structure of the μ-opioid receptor with PDB id 4DKL [39, 84]. TMs 5 and 6 of inactive A2AR and CB1R were modeled as observed on the structure of the μ-opioid receptor to facilitate the formation of the highly packed TM 5/6 interface. The A2AR and CB1R homodimers were modeled via the TM 4/6 interface, using the structure of CB2R with PDB id 5ZTY [85, 86]. The large outward movement of TM 6 for G protein coupling is not compatible with the TM 4/6 interface observed in this structure due to steric clashes. To avoid these clashes, the active protomer was manually rotated relative to the inactive protomer. The resulting tetrameric complex was refined using a 800-ns molecular dynamics simulation (as described in detail elsewhere [6]).

### [^14^C]glutamate release assay from striatal glutamatergic terminals

Male Wistar rats (180–240 g, 8-10 weeks old) were purchased from Charles-River (Barcelona, Spain), and housed with 12-h light on/off cycles under controlled temperature (23 ± 2 °C) and ad libitum access to food and water. Before brain extraction, the rats were deeply anesthetized with halothane (5%, 1 l/min flow rate; no reaction to tail pinch or handling, while still breathing). Experiments were carried out as previously described [19]. Briefly, the two striata were rapidly dissected in ice-cold Krebs’ solution (in mM: NaCl 132, KCl 3, KH_2_PO_4_ 1.2, MgSO_4_ 1.2, CaCl_2_ 2.5, NaHCO_3_ 25, glucose 5.5, HEPES 10; pH 7.4) and moved into 2 ml of ice-cold sucrose solution (0.32 M, containing 15 mM HEPES; pH 7.4) for homogenization with a Teflon homogenizer (Thomas Scientific, NJ, USA). The heavy debris particles in the homogenate were decanted at 1000*g* for 10 min at 4 °C, and the supernatant was saved. The first pellet (P1) was resuspended once again and centrifuged as above. Subsequently, the supernatants from the two centrifugations were pelleted at 20,000*g* for 30 min, to obtain the P2 crude synaptosomal fraction. The two P2 pellets (synaptosomes) from each rat were subsequently combined and stored on ice until use. For the assays with pertussis toxin (PTX), synaptosomes from each animal were resuspended in 2 ml of Krebs-HEPES solution, pregassed with 95% O_2_ and 5% CO_2_, containing PTX (2 μg/ml) and left tightly sealed at 4 °C for 4 h, then pelleted again for [^3^H]glutamate loading. Hereafter, all assay solutions contained the glutamate decarboxylase inhibitor, aminooxyacetic acid (100 μM), to prevent the transfer of [^3^H] labels to releasable molecules other than glutamate. Before the release experiments, synaptosomes from each animals were resuspended in 0.5 ml pregassed Krebs-HEPES solution and incubated in the presence of [^3^H]glutamate (specific activity, 60 Ci/mmol; final concentration, 200 nM; American Radiolabeled Chemicals Inc, Saint Louis, MO) for 15 min. When necessary, incubations with TM5 (20 μM) or with TM7 (20 μM) of A2AR occurred also during this period. Subsequently, a 8-microvolume chamber superfusion setup was filled with preloaded synaptosomes (chambers/rat) which were trapped by layers of Whatman GF/B filters (Sigma-Aldrich). Synaptosomes were superfused continuously at a rate of 0.8 ml/min with pre-gassed Krebs-HEPES solution (37 °C) until the end of the experiments. Upon termination of a 10-min washout and after collecting three 2-min samples as baseline, neurotransmitter release was stimulated twice with 30 mM KCl (S_1_, S_2_) with 10-min interval (Fig. 6a). The CB1R agonist WIN55,212-2 (Tocris Bioscience, Bristol, UK), A_2A_R ligands (Tocris Bioscience), or their solvent, DMSO (0.1% v/v), were added to the superfusion medium 4 min before S_2_ and continued to be present until the end of the experiment (that is, there was no washout study).

Treatments were performed in duplicate (i.e., 1 pair of control *versus* 3 pairs of different treatments per animal), and the intra-chamber S_2_/S_1_ ratios served to evaluate the effect of drug treatments and expressed as % of DMSO control (Fig. 6a). All data are represented as means ± S.E.M. of “*n* ≥ 5” observations (rats) in duplicates. As control S_2_/S_1_ ratios do not have biological significance, they were taken as 100% in each experiment, and treatment S_2_/S_1_ ratios were normalized to them. Normalized data were then tested for normal distribution by the Kolmogorov-Smirnov test, and subsequently analyzed with one-sample *t*-test against the hypothetical value of 100 (%), and *p* < 0.05/3 (a correction factor for three treatments using one control) was accepted for significant difference. Tests were performed using the GraphPad Prism 5.0 software.

## Supplementary information

**Additional file 1: Figure S1.** G protein coupling of A2AR and CB1R in the A2AR-CB1R heteromer. **Figure S2.** Lack of modulation by the CB1R agonist CP55940 on A2AR-mediated Gs protein activation in the A2AR-CB1R heteromer. **Figure S3.** Tetrameric structure of A2AR-CB1R heteromer. **Figure S4.** Gs-dependent A2AR-mediated modulation and Gi-dependent CB1R-mediated modulation of AC signaling in HEK-293T cells. **Figure S5.** Control of striatal glutamate release by A2AR-CB1R and A1R-A2AR heteromers.

**Additional file 2.** Data values 1. EC_50_ values from Additional file 1: Figure S1. Data values 2. EC_50_ and E_max_ values from Additional file 1: Figure S2. Data values 3. Values of cAMP formation from Fig. 5. Data values 4. Values of constitutive activation (cAMP formation) from Fig. 7.

## Data Availability

All data generated or analyzed during this study are included in this published article and its supplementary information files (Additional files 1 and 2). The crystal structures 5NM4, 5G53, 6GDG, 5UO9, 6N4B, 4DKL, and 5ZTY, used to build the computational models, are available from Protein Data Bank (PDB; http://www.rcsb.org; specific PDB entries included in the reference list).
