# Supplementary material for: Control of glutamate release by complexes of adenosine and cannabinoid receptors
Source: BMC Biol. 2020 Jan 23;18:9. doi: 10.1186/s12915-020-0739-0 (PMC6979073; doi:10.1186/s12915-020-0739-0)
Supplement: Supplementary file 1 — Additional file 1: Figure S1. G protein coupling of A2AR and CB1R in the A2AR-CB1R heteromer. Figure S2. Lack of modulation by the CB1R agonist CP55940 on A2AR-mediated Gs protein activation in the A2AR-CB1R heteromer. Figure S3. Tetrameric structure of A2AR-CB1R heteromer. Figure S4. Gs-dependent A2AR-mediated modulation and Gi-dependent CB1R-mediated modulation of AC signaling in HEK-293T cells. Figure S5. Control of striatal glutamate release by A2AR-CB1R and A1R-A2AR heteromers. [file 12915_2020_739_MOESM1_ESM.docx]

**Additional file 1**

**
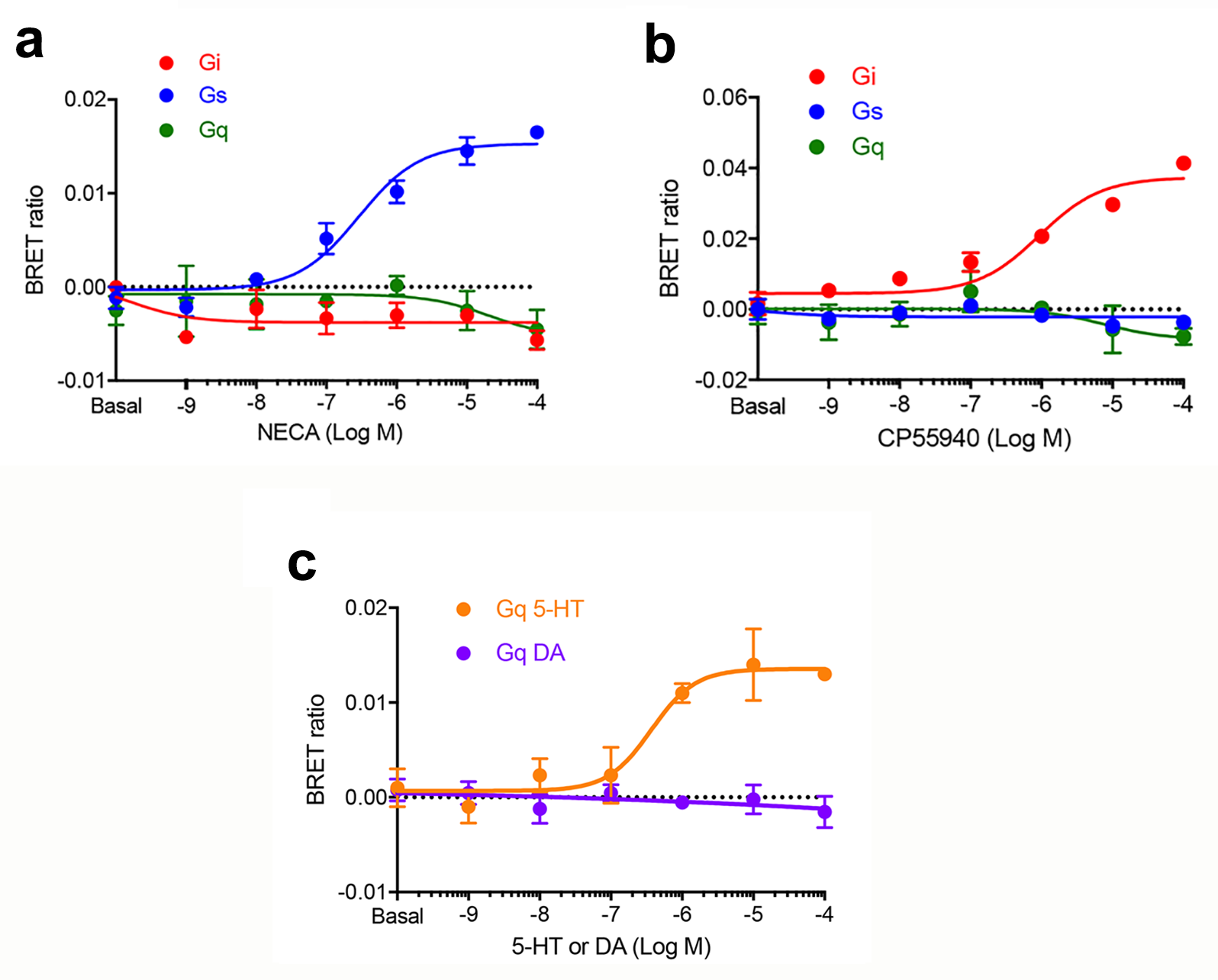
**

**Fig. S1. G protein coupling of A2AR and CB1R in the A2AR-CB1R heteromer. a-b** CODA-RET experiments, where two complementary halves of Rluc (cRluc and nRluc) are respectively fused to the A2AR and CB1R and YFP is fused to the α subunit of Gi, Gs or Gq. HEK-293T cells were transiently transfected with cDNAs of A2AR-cRluc (3.33 μg), CB1R-nRluc (1.67 μg), Gαi1-YFP (5 μg), Gαs-YFP (5 μg) or Gαq-YFP (5 μg) and non-fused β1 and γ2 subunits (4.5 μg and 5 μg, respectively). **c** CODA-RET experiments, where cRluc and nRluc are respectively fused to the 5-HT2AR and D2R and YFP is fused to the α subunit of Gq. HEK-293T cells were transiently transfected with cDNAs of A2AR-cRluc (3.33 μg) and CB1R-nRluc (1.67 μg) or 5-HT2AR-cRluc (1.67 μg) and D2R-nRluc (3.33 μg) and Gαi1-YFP (5 μg), Gs-YFP (5 μg) or Gαq-YFP (5 μg) and non-fused β1 and γ2 subunits (4.5 μg and 5 μg, respectively). Graphs represent concentration-response curves of the effect of the non-selective adenosine receptor agonist NECA (**a**), the selective CB1R agonist CP55940 (**b**), and serotonin (5-HT) and dopamine (DA) (**c**) on the ligand-induced BRET changes, which are determined by changes in the interaction of the A2AR-CB1R heteromer with Gi, Gs and Gq proteins (red, blue and green plots, respectively) or the 5-HT2AR-D2R heteromer with Gq. Data are means ± S.E.M. of triplicate BRET ratio values of a representative experiment (see text for EC_50_ values of 5 independent experiments).

**
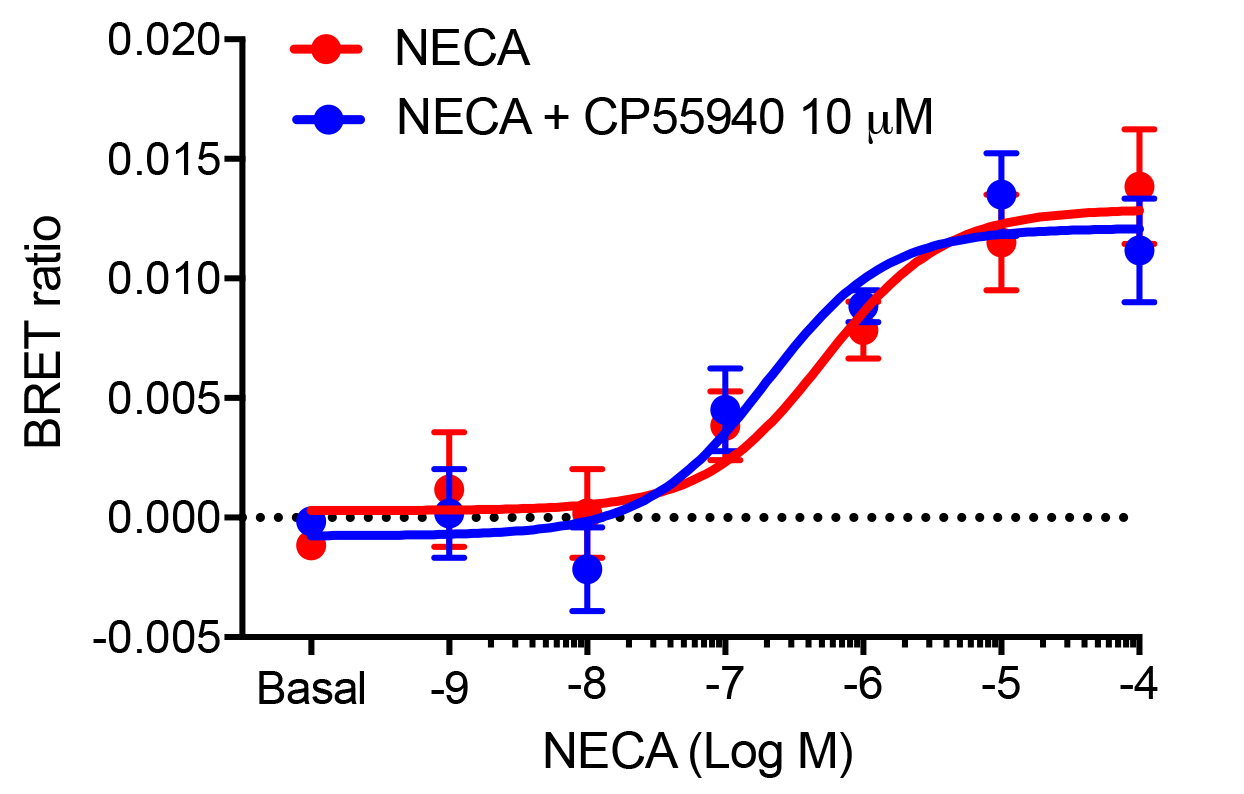
**

**Fig. S2.** **Lack of modulation by the CB1R agonist CP55940 on A2AR-mediated Gs protein activation in the A2AR-CB1R heteromer.** CODA-RET experiments, where two complementary halves of Rluc (cRluc and nRluc) are respectively fused to the A2AR and CB1R and YFP is fused to the α subunit of Gs. HEK-293T cells were transiently transfected with cDNAs of A2AR-cRluc (3.33 μg), CB1R-nRluc (1.67 μg), Gαs-YFP (5 μg) and non-fused β1 and γ2 subunits (4.5 μg and 5 μg, respectively). Concentration-response curves of the effect of the non-selective adenosine receptor agonist NECA on the ligand-induced BRET changes, which are determined by changes in the interaction of the A2AR-CB1R heteromer with Gs, in the presence (blue plot) and absence (red plot) of the selective CB1R agonist CP55940 (10 μM). See text for the analysis of EC_50_ and E_max_ values from 5 independent experiments.

**
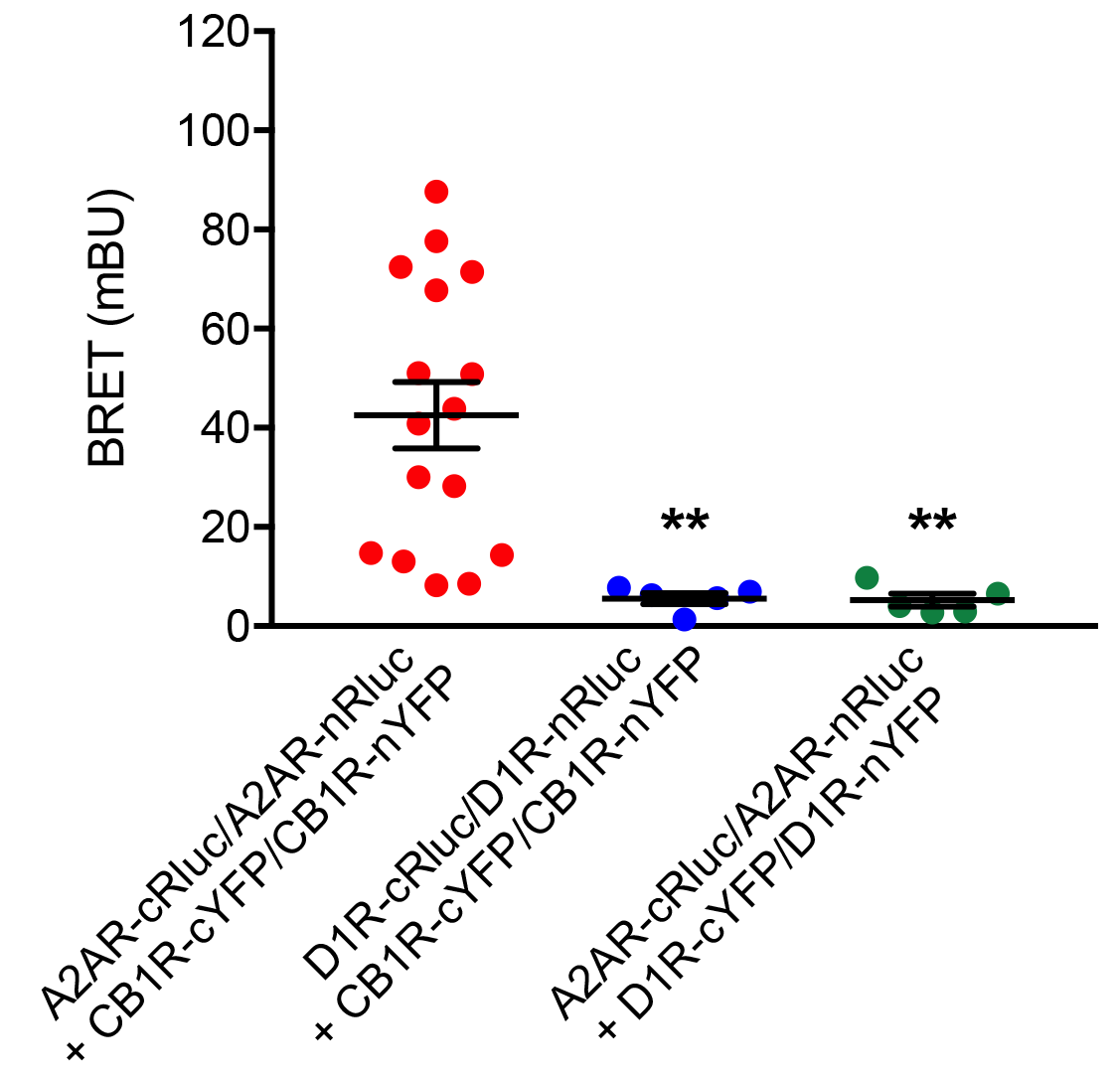
**

**Fig. S3. Tetrameric structure of A2AR-CB1R heteromer.** BRET experiment with double complementation of both BRET biosensors. HEK-293T cells were transiently transfected with cDNAs of two different molecules of A2AR separately fused to complementary halves of Rluc (A2AR-cRluc and A2AR-nRluc; 0.5 μg in both cases) and two different molecules of CB1R separately fused to complementary halves of YFP (CB1R-cYFP and CB1R-nYFP; 3 μg in both cases). cDNAs of D1R-cYFP and D1R-nYFP (0.25 μg in both cases) and D1R-cYFP and D1R-nYFP (3.5 μg in both cases) were also used as controls. Values are means ± S.E.M. (n = 5-16 with triplicates) of milli BRET units (mBU; net BRET x 1,000) and analyzed statistically with one-way ANOVA, followed by Dunnett’s multiple comparison test. Significant BRET values were obtained with co-transfection of A2AR-cRluc, A2AR-nRluc, CB1R-cYFP and CB1R-nYFP, compared with controls (**: p < 0.01), indicating the formation of oligomers of A2AR and CB1R homodimers.

**
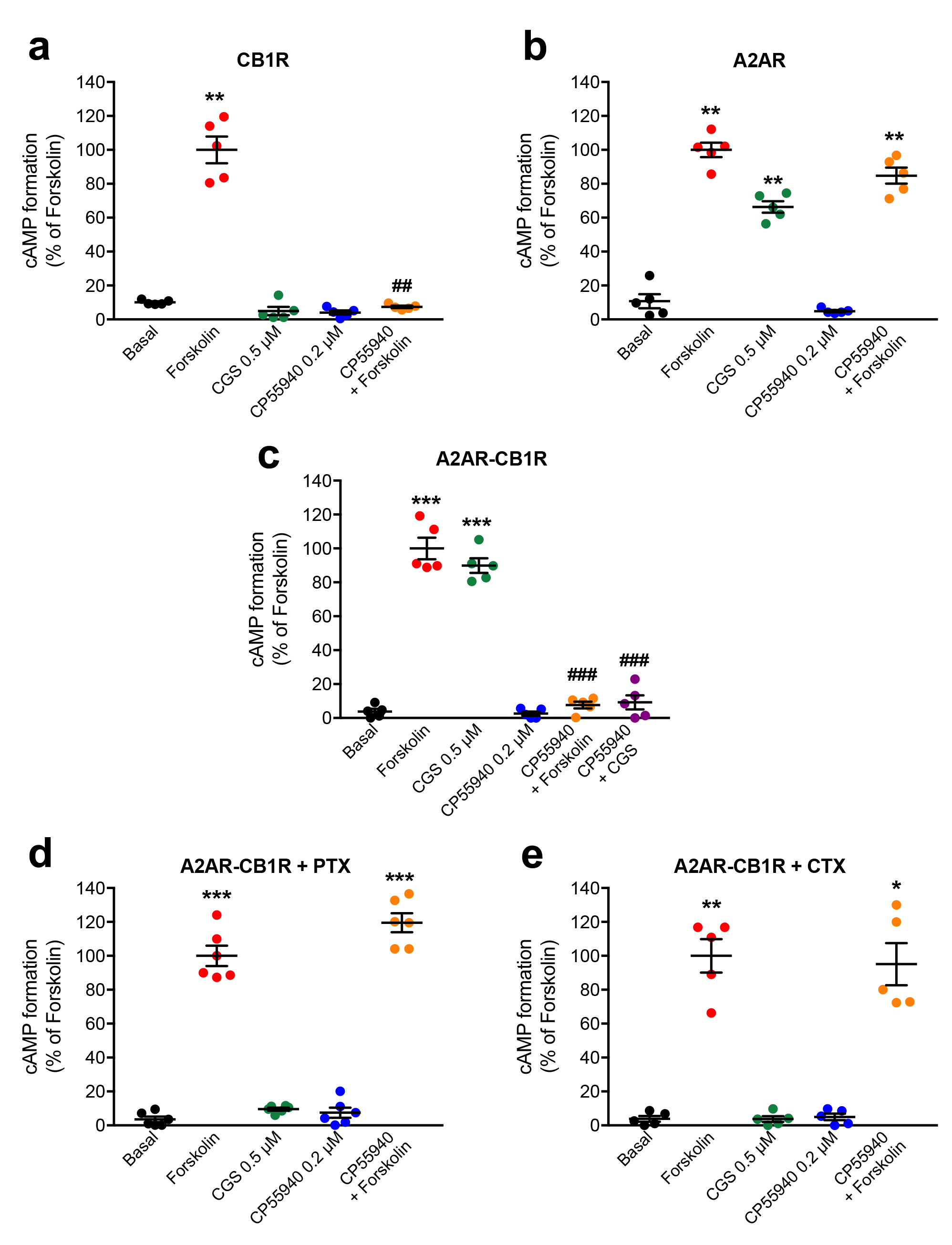
**

**Fig. S4. Gs-dependent A2AR-mediated modulation and Gi-dependent CB1R-mediated modulation of AC signaling in HEK-293T cells. a-e** cAMP formation induced by forskolin (500 nM) or by the A2AR agonist CGS21680 (CGS, 500 nM) and counteractive effects of the CB1R agonist CP55940 (200 nM). HEK-293T cells were transiently transfected with cDNAs of CB1R (**a**; 1.25 μg), A2AR (**b**; 1.25 μg) or with both (**c-e**; 2 μg in both cases) in the absence of toxins (**c**) or pre-treated overnight with pertussis toxin (PTX; 10 ng/ml; **d**) or for 2h with cholera toxin (CTX; 100 ng/ml; **e**). Values are expressed as means ± S.E.M. (n = 5-6 with triplicates) of the percentage of forskolin-induced cAMP formation and analyzed statistically with repeated measures ANOVA, followed by Dunnett’s multiple comparison test (** and ***: p < 0.01 and p < 0.001, respectively, compared with basal; **^##^** and **^###^**: p < 0.01 and p < 0.001, respectively, compared to forskolin or CGS).


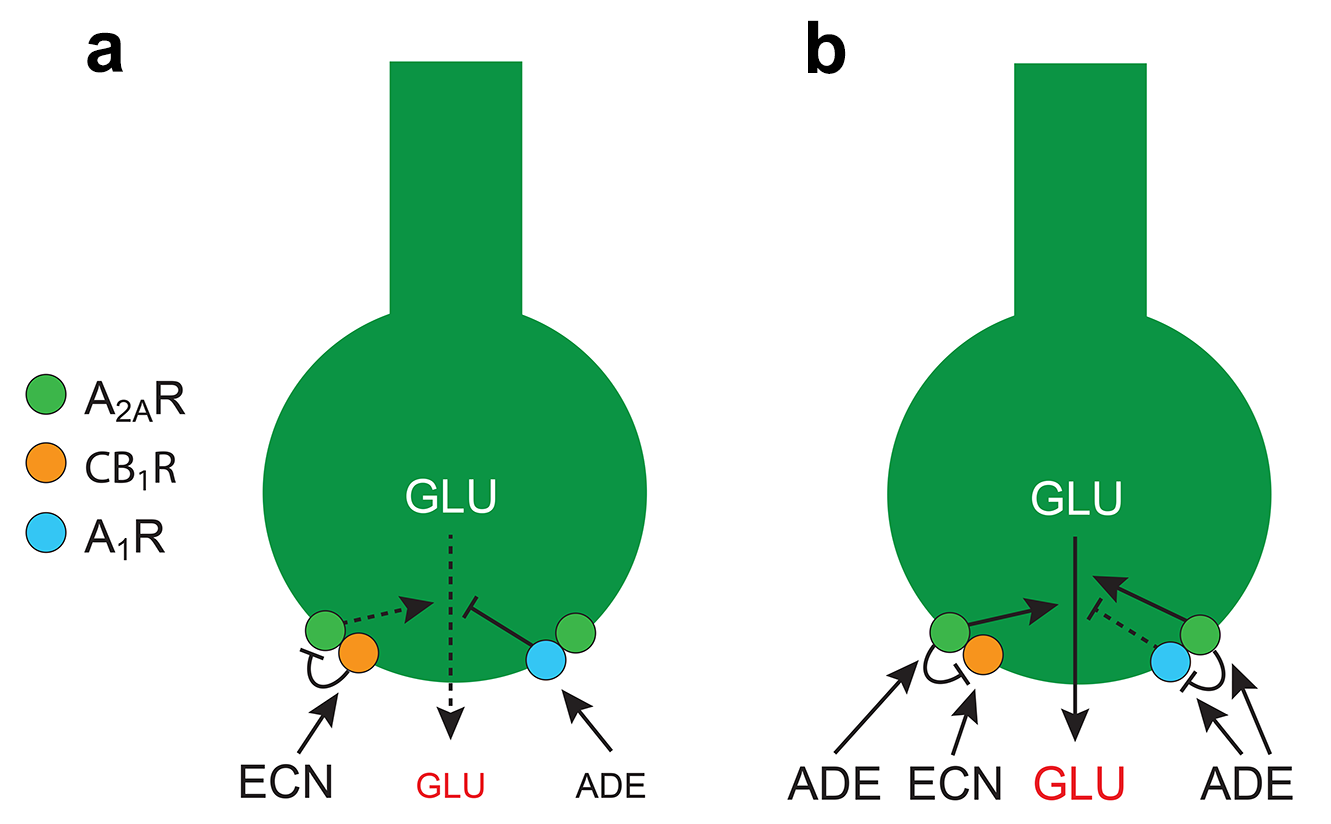


**Fig. S5.** **Control of striatal glutamate release by A2AR-CB1R and A1R-A2AR heteromers**. Schematic representation of corticostriatal glutamatergic terminals and the modulatory role of adenosine (ADE) and endocannabinoids (ECN) on glutamate (GLU) release mediated by A2AR-CB1R and A1R-A2AR heteromers. **a** Under low extracellular concentrations of ADE, the predominant activation of A1R within the A1R-A2AR heteromer leads to a predominant inhibition of GLU release, which can be further inhibited by ECN, which counteract A2AR-mediated constitutive activity within the A2AR-CB1R heteromer by means of the canonical Gi-Gs antagonistic interaction at the AC level. **b** Under high extracellular concentrations of ADE, the activation of A2AR leads to the allosteric counteraction of A1R- and CB1R-mediated activation within the A1R-A2AR and A2AR-CB1R heteromers and to the A2AR-Gs-AC mediated activation of GLU release.
